# A malectin domain kinesin functions in pollen and seed development in Arabidopsis

**DOI:** 10.1093/jxb/eraa023

**Published:** 2020-01-17

**Authors:** Sergio Galindo-Trigo, Thomas M Grand, Christian A Voigt, Lisa M Smith

**Affiliations:** 1 Department of Animal and Plant Sciences and The Plant Production and Protection (P3) Centre, University of Sheffield, Western Bank, Sheffield, UK; 2 University of Essex, UK

**Keywords:** Arabidopsis, cell division, kinesin, malectin domain, pollen development, seed development

## Abstract

The kinesin family is greatly expanded in plants compared with animals and, with more than a third up-regulated in expression during cell division, it has been suggested that this expansion facilitated complex plant-specific cytoskeletal rearrangements. The cell cycle-regulated kinesins include two with an N-terminal malectin domain, a protein domain that has been shown to bind polysaccharides and peptides when found extracellularly in receptor-like kinases. Although malectin domain kinesins are evolutionarily deep rooted, their function in plants remains unclear. Here we show that loss of MALECTIN DOMAIN KINESIN 2 (MDKIN2) results in stochastic developmental defects in pollen, embryo, and endosperm. High rates of seed abnormalities and abortion occur in *mdkin2* mutants through a partial maternal effect. No additive effect or additional developmental defects were noted in *mdkin1 mdkin2* double mutants. MDKIN2 is expressed in regions of cell division throughout the plant. Subcellular localization of MDKIN2 indicates a role in cell division, with a possible secondary function in the nuclei. Our results reveal a non-essential but important role for a malectin domain kinesin during development in plants.

## Teaser

MALECTIN DOMAIN KINESIN 2 (MDKIN2) functions in the development of pollen grains, embryos, and endosperm. Subcellular protein localization of MDKIN2 indicates a potential role in cell division.

## Introduction

In plants, a diverse array of kinesins act as microtubule-based molecular motors that mediate core biological processes including cell division, intracellular transport, and cell shape determination (Zhu and Dixit, 2012). Expansion of the kinesin family in plants (to 61 family members in Arabidopsis) and the number of kinesins without homologues in animals suggest functional divergence of plant kinesins, which is probably driven by the differences in microtubule rearrangements during mitosis and cytokinesis in plants compared with animals (Reddy and Day, 2001; Richardson *et al.*, 2006; Zhu and Dixit, 2012). Plant-specific functions may relate to cytoskeletal rearrangements such as formation of the preprophase band and phragmoplast, a hypothesis which is supported by transcriptional up-regulation and specific localization patterns of many plant kinesins during mitosis (Menges *et al.*, 2005; Vanstraelen *et al.*, 2006; Miki *et al.*, 2014). However, the function of most kinesins remains unknown, impairing understanding of how cell division is accomplished in plants. With the possibility that kinesins may act at multiple stages of the cell cycle and synergistically or antagonistically with each other, determination of their functions at every stage of the cell cycle becomes more complex (Zhu and Dixit, 2012).

Cell division is a fundamental biological process of key importance in the development of all multicellular organisms. During cell division, the cytoskeleton, consisting of microtubules and actin filaments in plants, undergoes extensive rearrangement to coordinate the movement of chromosomes and organelles, and position the new cell wall. At the start of mitosis, rearrangement of microtubule arrays leads to formation of a transient preprophase band at the cell cortex which functions in positioning of the spindle around the pre-mitotic nucleus and orientation of the phragmoplast (Murata and Wada, 1991; Ambrose and Cyr, 2008). In plants, the mitotic spindles lack centrosomes, which are replaced by diffuse spindle poles consisting of the minus-ends of microtubule arrays (Wadsworth *et al.*, 2011). The midzone is formed during anaphase in the region defined by overlapping plus-ends of microtubules and helps generate the forces needed for spindle elongation. In late mitosis (telophase and cytokinesis), the midzone defines the plane in which the phragmoplast expands towards the cortex and guides cell plate construction to meet the extant cell wall at the site of the former preprophase band (Müller and Jürgens, 2016). Kinesins function during many stages of cell division including a possible role in cell plate construction through transportation of Golgi-derived vesicles (Kong *et al.*, 2015). Given the extensive cytoplasmic rearrangements, it is perhaps unsurprising that at least a third and possibly as many as two-thirds of kinesins are implicated in mitosis (Vanstraelen *et al.*, 2006; Miki *et al.*, 2014).

Through cross-linking of cellular components with the cytoskeleton, kinesins facilitate microtubule organization via microtubule bundling and polymerization dynamics, and movement of cellular components along the microtubules via directional transport. The kinesins use ATP hydrolysis to drive locomotion along microtubules (or in some cases to depolymerize microtubules) (Sharp *et al.*, 2000). While the ATP hydrolysis, microtubule-binding, and motor domains are conserved between kinesins in plants and animals, the tail domains that bind specialized cargos are highly divergent even within a species, varying in the protein domains present (Reddy and Day, 2001; Miki *et al.*, 2005).

Among the plant kinesins, the large kinesin-14 family with 21 members and six subfamilies in Arabidopsis is notable for the presence of various plant-specific tail domains (Richardson *et al.*, 2006). This family is also unusual in that the microtubule-binding domain and kinesin motor domain are often internal or C-terminal, and some family members have been shown to move towards the minus- rather than the plus-ends of the microtubules, thus facilitating retrograde transport (i.e. transport towards the nucleus; Cross, 2010; Jonsson *et al.*, 2015). This polarity of movement is determined by the amino acid sequences in the neck regions surrounding the kinesin domain (Endow, 1999). Retrograde movement has led to the suggestion that the kinesin-14 family in plants may have homologous roles to the animal dyneins in spindle formation and positioning (Zhu and Dixit, 2012).

The kinesin-14 family includes many members that are up-regulated during mitosis and function during cell division (Menges *et al.*, 2005; Vanstraelen *et al.*, 2006; Miki *et al.*, 2014). Rice KHC1 functions in pre-mitotic nuclear migration (Frey *et al.*, 2010), while ATK1 and Arabidopsis KINESIN-LIKE CALMODULIN-BINDING PROTEIN (KCBP) bind to the preprophase band that marks the future cell division plane (Bowser and Reddy, 1997; Marcus *et al.*, 2003). KCBP also acts in organization of the cortical microtubules, and functions in spindle and phragmoplast formation (Bowser and Reddy, 1997; Mathur and Chua, 2000; Vos *et al.*, 2000). Dual binding of microtubules and actin filaments by KCBP is key in configuration of the cytoskeleton during trichome cell morphogenesis (Tian *et al.*, 2015). Rice ATK1 is required for spindle morphogenesis (Chen *et al.*, 2002; Marcus *et al.*, 2002) and, along with KCBP, localizes to interphase and mitotic microtubule arrays (Lipka and Müller, 2012). Other characterized kinesin-14s have defined roles in cross-linking antiparallel microtubules at the spindle and pulling the two halves of the spindle towards each other (Ambrose *et al.*, 2005). The kinesin-14 KCH has dual localization, being found at the preprophase band and the phragmoplast in tobacco protoplasts (Klotz and Nick, 2012). Away from cell division, AtKP1 localizes to the mitochondria (Ni *et al.*, 2005), and KCA1 and KCA2 function in chloroplast movement (Suetsugu *et al.*, 2010). Mutation of some kinesin-14 family members results in developmental defects, with KCBP having a trichome phenotype (Mathur and Chua, 2000). Thus, many of the characterized kinesin-14 family members act during cell division or in localization of plastids, with some also influencing plant development.

Here we report on a kinesin-14 family member in Arabidopsis that contains a malectin domain, a protein fold most frequently associated with receptor-like kinases where it binds peptides and carbohydrates (Haruta *et al.*, 2014; Ge *et al.*, 2017; Stegmann *et al.*, 2017; Feng *et al.*, 2018; Gonneau *et al.*, 2018; Lin *et al.*, 2018, Preprint). Given the importance of the malectin domain receptor-like kinases in plant development, we decided to examine whether the malectin domain kinesins also function in development. Moss malectin domain kinesin homologues localize to the midzone, intraspindle, and polar region microtubules during mitosis (Miki *et al.*, 2014), indicating a potential role for this group of proteins in cell division. Although Arabidopsis MALECTIN DOMAIN KINESIN 2 (MDKIN2) is expressed in diverse plant tissues undergoing cell division, defects are only evident during seed and pollen development. The seed phenotype is partially due to a maternal effect, with no functional redundancy evident between MDKIN2 and the second Arabidopsis malectin domain kinesin, MDKIN1. The localization pattern of MDKIN2 during cell division is similar to that reported for related moss proteins, with the notable exception that MDKIN2 is localized to the nucleus during interphase. The mutant phenotype combined with the localization pattern of the protein argues for an important but non-essential role for the kinesin during cell division.

## Materials and methods

### Plant lines

Four T-DNA insertion lines in *Arabidopsis thaliana At2g22610* (SALK_063550, SALK_072997, SAIL_312_D02, and SALK_049342; referred to hereafter as *mdkin2-1* to *mdkin2-4*, respectively), and one T-DNA line for *At1g72250* (SALK_025966; *mdkin1-1*) were ordered from the Nottingham Arabidopsis Stock Centre (NASC; Sessions *et al.*, 2002; Alonso *et al.*, 2003). The lines were PCR genotyped to identify plants with homozygous T-DNA insertions (for primers, see Supplementary Table S1 at *JXB* online). Quantitative reverse transcription–PCR (RT–qPCR) was performed by extracting RNA from leaf tissue of three plants per triplicate sample using a Sigma Spectrum™ Plant Total RNA kit. A 1 µg aliquot of RNA was DNase I treated and cDNA was synthesized with hexamer primers (Thermo Scienctific RevertAid kit). qPCRs were performed with the Qiagen SYBR Green PCR kit using 20 s extension time per cycle and an annealing and extension temperature of 60 °C (for primers, see Supplementary Table S2). Accession Col-0 was used as a wild-type control. A plant line expressing *pUBQ::mRFP-βTUBULIN6* was also acquired from the NASC (N67065; Ambrose *et al.*, 2011).

Plants were grown in a 4:1 mix of Levington M3 compost:sand in a controlled-environment chamber (Conviron) with 22 °C constant temperature, 60% humidity, and 16 h light/day at ~120 µmol s^−1^ m^−2^. For β-glucuronidase (GUS) staining of seedlings and epifluorescence microscopy of roots, seeds were surface sterilized with chlorine gas, placed on 0.5× Murashige and Skoog medium plates (Murashige and Skoog Basal Salt Mixture; Sigma) with 0.8% (w/v) plant agar (Duchefa Biochemie) pH 5.7, and stratified for 2–4 d before growth in a Snijders Scientific cabinet at a constant 22 °C and 16 h light/day.

### Phenotyping

To examine seed development, mature siliques were opened along the junction of the valves and replum using Dumont no. 5 forceps, valves were opened onto double-sided tape, and seeds were photographed on a lightbox. Seeds were scored according to whether the ovule appeared unfertilized (small and white), seed development had been aborted early, and the seed was abnormal in shape/size or was of normal shape and size. Embryo and endosperm development were assessed by clearing seeds in a 2:1 dilution of Hoyer’s solution as per Bayer *et al.* (2009) before imaging on an Olympus BX51 microscope.

Pollen development was visualized via bright field microscopy on a Leica DM6 microscope and by a Hitachi TM3030Plus benchtop scanning electron microscope. The diameter of pollen grains was measured from bright field images of five developing anthers per line using ImageJ. To test whether pollen grain size distributions were affected by genotype, we applied the following steps. First, pollen grain size was normalized to the median value for each anther to adjust for different stages of anther development. Secondly, the number of pollen grains smaller than a nominal size threshold of 2 SDs below the normalized mean of Col-0 was calculated for each genotype. Finally, a Fisher’s exact test was applied to each genotype to test whether a greater number of pollen grains than expected fell below this threshold, and the *P*-values were corrected for multiple testing. Contrast of bright field images was adjusted in Inkscape. Pollen viability was assessed by fluorescein diacetate (FDA) staining as per Chang *et al.* (2014) before imaging on an Olympus BX51 microscope with LED excitation at 400 nm and a U-MNV2 cube (455 nm longpass filter).

To determine the survival of seeds hemizygous and homozygous for the *mdkin2-1* T-DNA insertion, a confirmed homozygous *mdkin2-1* mutant was crossed to a Col-0 plant. F_2_ seedlings were scored for zygosity by DNA extraction and PCR amplification of the wild-type and mutant alleles. Parental effects were assessed through reciprocal and selfed crosses between Col-0 and *mdkin2-1* plants. Siliques were dissected at 10–14 d post-fertilization, and seed development was scored from photographed siliques.

### Expression and localization

To examine *MDKIN2* expression at the tissue level, a 1 kb fragment directly upstream of the *MDKIN2* translational start site was amplified using Phusion DNA polymerase (NEB), cloned into pCR8 (ThermoFisher), the sequence was verified and then subcloned into *pGWB433* 5' of the *GUS* reporter gene using LR clonase (ThermoFisher; see Supplementary Table S3 for primers). The construct was transformed into Col-0 plants by floral dip. T_1_ seedlings were selected on plates using kanamycin. GUS staining was performed by fixing tissue in 90% acetone on ice for 20 min then incubation with X-Gluc (Melford Biolaboratories Ltd) as per Weigel and Glazebrook (2002) before light microscopy with a Leica M165FC or Olympus BX51 microscope. At least eight T_1_ plants were examined to identify representative patterns of expression

To examine subcellular localization of MDKIN2, the 1 kb promoter region and full protein-coding sequence were fused to *tdTomato*, *mCherry*, and *GFP* (green fluorescent protein) reporter genes in a pGreenII (Basta resistance) plasmid (Mathieu *et al.*, 2007; see Supplementary Table S3 for primers). The fluorescent protein reporter constructs were transformed into *mdkin2-1*, and complementation of the *mdkin2-1* phenotype was assessed by seed development and pollen grain size. The pMDKIN2::MDKIN2-FP lines were crossed to pUBQ1::mRFP-βTUB and pHTR12::HTR12-mCherry plants to examine localization of MDKIN2 compared with tubulin and centromeric histones, respectively. Subcellular localization of MDKIN1 was determined by fusing a 2.2 kb promoter region and full protein-coding sequence to an *mCitrine* reporter as per MDKIN2 (see Supplementary Table S3 for primers).

Epifluorescence microscopy was performed using a Leica DM6 or Olympus BX51 microscope with HC PL Fluotar objectives ×20, 0.55 NA, ×40, 0.80 NA, and ×100, 1.32 NA (oil immersion) or UPlanFl ×20, ×40 (dry), and ×100 (oil immersion) objectives, respectively, and LED illumination at 470 nm or 535 nm. Image brightness and contrast were adjusted for each channel in ImageJ prior to merging channels and figure assembly in Adobe Illustrator. Point spread functions were calculated with the Diffraction PSF 3D plugin and applied to the images using the Iterative Deconvolve 3D plugin in ImageJ (Dougherty, 2005).

Spinning disc confocal microscopy was performed with a Perkin Elmer Ultraview VoX system with an inverted Olympus IX81 microscope (UplanSApo ×100 oil NA 1.4 and excitation with 488 nm or 561 nm lasers). *Z*-stacks were acquired from areas containing structures of interest revealing combined MDKIN2–GFP/mRFP–βTUB and MDKIN2–GFP/HTR12–mCherry expression, respectively, at different cell cycle stages. For 3D rendering and high-resolution localization of MDKIN2 compared with tubulin and centromeric histones, respectively, at defined cell cycle stages, cellular structures of interest were cropped from initial *Z*-stacks using the rectangle selection tool of the ImageJ image processing software (ImageJ 1.52p, National Institutes of Health, USA). From cropped *Z*-stacks, focal planes above and below the structure of interest containing unspecific fluorescence signals were removed to allow the highest possible protein localization via 3D rendering. *Z*-stacks were further processed using the Surpass tool with default settings of the Imaris image processing package version 7.7.2 (Bitplane, Oxford Instruments, Zurich, Switzerland) for final 3D rendering. The integrated Animation software tool was used to generate rotation movies of 3D-rendered *Z*-stacks including cellular structures of interest. In dual-colour images as well as movies revealing combined MDKIN2–GFP/mRFP–βTUB and MDKIN2–GFP/HTR12–mCherry expression, green colour was assigned to MDKIN2–GFP and magenta colour to mRFP–βTUB and HTR12–mCherry.

### Bioinformatics

Protein identity and similarity were calculated for MDKIN1 and MDKIN2 by aligning the amino acid sequences of At1g72250.1 and At2g22610.1 in LALIGN (Huang and Miller, 1991). Homologues of *MDKIN1* and *MDKIN2* in *Arabidopsis lyrata* and *Capsella rubella* were identified via NCBI BLAST (Altschul *et al.*, 1997), and pairwise Ka/Ks calculated through using Kimura’s two-parameter (K2P) model (Zhang *et al.*, 2006). Sequences for *MDKIN1* and *MDKIN2* from 1135 Arabidopsis accessions were downloaded from www.1001genomes.org (1001 Genomes Consortium, 2016), then sequences with more than one consecutive N and identical sequences were removed to leave a non-redundant set of high-confidence nucleotide sequences. Gene sequences (123 for *MDKIN1* and 160 for *MKDIN2*) were aligned using Pearson/FASTA output in MUSCLE (Edgar, 2004) to generate identity matrices, then the average identity per nucleotide position was calculated for the genes. A phylogenetic tree of MDKIN sequences from multiple species was built using full-length sequences of MDKINs aligned using ClustalX (Larkin *et al.*, 2007) before a Neighbor–Joining phylogenetic tree was generated with MEGA5.2 (Saitou and Nei, 1987; Tamura *et al.*, 2011). Sequence data were downloaded through Phytozome, including the liverwort *Marchantia polymorpha* (Sasaki *et al.*, 2007; these sequence data were produced by the US Department of Energy Joint Genome Institute http://www.jgi.doe.gov/ in collaboration with the user community); the model bryophyte *Physcomitrella patens* (Rensing *et al.*, 2008; Lehti-Shiu and Shiu, 2012); the lycophyte *Selaginella moellendorffii* (Banks *et al.*, 2011; Lehti-Shiu and Shiu, 2012); the conifer *Picea abies* (Nystedt *et al.*, 2013); and *Amborella trichopoda* (Amborella Genome Project, 2013). Nuclear localization signals were predicted with NLS Mapper using with a 5.0 cut-off score (nls-mapper.iab.keio.ac.jp; Kosugi *et al.*, 2009). *MDKIN2* expression from microarray data was viewed in ePlant (Fucile *et al.*, 2011).

### Statistical analysis

Statistical significances of categorical phenotypic distributions were compared using Pearson’s χ ^2^ tests, assuming that wild-type Col-0 plants have the expected distribution. Pollen grain size distributions were compared by Fisher’s exact test.

## Results

### MDKIN proteins have deep evolutionary roots

Two atypical members of the kinesin-14 family in Arabidopsis encode a malectin domain in their N-terminal regions. This domain, which has been named after its structural similarity to a carbohydrate-binding protein found in animals (Schallus *et al.*, 2008), is most frequently associated with receptor-like kinases in plants. The Arabidopsis MDKINs are highly diverged with only 47.9% identity and 73.4% similarity in the 794 amino acids that align between MDKIN1 (1195 amino acids) and MDKIN2 (1083 amino acids), suggesting either a rapid rate of divergence due to positive selection, a deep-rooted gene duplication event, or a combination of the two.

The plant-specific protein domain configuration of a malectin domain paired with a central kinesin domain is present in dicots, monocots, and early vascular plants such as a lycophyte (*S. moellendorffii*), a moss (*P. patens*), and a liverwort (*M. polymorpha*). Given that the last common ancestor of both liverworts and angiosperms is placed at ~475 million years ago (Mya) (Delwiche and Cooper, 2015), the conservation of this domain configuration across a long evolutionary time scale suggests that MDKINs may have an integral role in plants. A phylogenetic tree of MDKIN proteins from key species indicates that there are two major clades of the protein (Supplementary Fig. S1), with one member from Arabidopsis in each clade. These clades arose before the last common ancestor of Arabidopsis and *A. trichopoda*, an extant member of the earliest divergent angiosperm lineage, which last shared a common ancestor with Arabidopsis at least 160 Mya (Amborella Genome Project, 2013), indicating a deep-rooted duplication of *MDKIN* genes. Thus, duplication of the *MDKIN* genes occurred around the divergence of gymnosperms and angiosperms. We hypothesize that while MDKIN1 probably has an evolutionarily conserved role in development across all land plants, the MDKIN2 variant could have been recruited to angiosperm-specific developmental processes and thus become fixed within this lineage.

Whether *MDKIN* genes are under positive selection, thus driving rapid divergence in the genomic sequences, was assessed by calculating Ka/Ks for Arabidopsis *MDKIN1* and *MDKIN2* compared with homologues from *A. lyrata* and *C. rubella*. Ka/Ks is a measure of purifying selection where a ratio of non-synonymous to synonymous mutations of >1 indicates positive selection. For *MDKIN1*, Ka/Ks was 0.66 and 0.65 for the pairwise comparisons with *A. lyrata* and *C. rubella*, respectively, while Ka/Ks was 0.57 for each pairwise comparison for *MDKIN2*, indicating possible stabilizing selection rather than positive selection. We also examined nucleotide divergence in the coding regions amongst Arabidopsis accessions to understand whether the *MDKIN* genes are undergoing a rapid rate of divergence within our study species. Extensive resequencing has led to the availability of many high-quality genomes of this model species (Cao *et al.*, 2011). Coding regions that contain contiguous ambiguous nucleotides were excluded from our analysis, as were identical nucleotide sequences that probably arise from very closely related accessions. Nucleotide identity was 99.7% for *MDKIN1* and 99.6% for *MDKIN2* genomic regions, rates which are similar to the average of ~99.5% nucleotide diversity for all Eurasian Arabidopsis genomes (1001 Genomes Consortium, 2016). Thus, divergence between the two *MDKIN* genes and proteins is likely to be due to a deep-rooted gene duplication event, with different functions for the two MDKIN clades hypothesized. While both MDKIN proteins have an N-terminal malectin domain and a central kinesin domain, MDKIN1 has a single low confidence nuclear localization signal as opposed to the two nuclear localization signals in MDKIN2 (one high confidence and one low confidence). Therefore, the function of these two MDKIN proteins may be determined in part by different subcellular localizations as MDKIN1 is predicted to have a dual nuclear and cytoplasmic localization while MDKIN2 is predicted to be predominantly nuclear (Kosugi *et al.*, 2009).

### MDKIN2 loss affects seed and pollen development

**Fig. 1.**
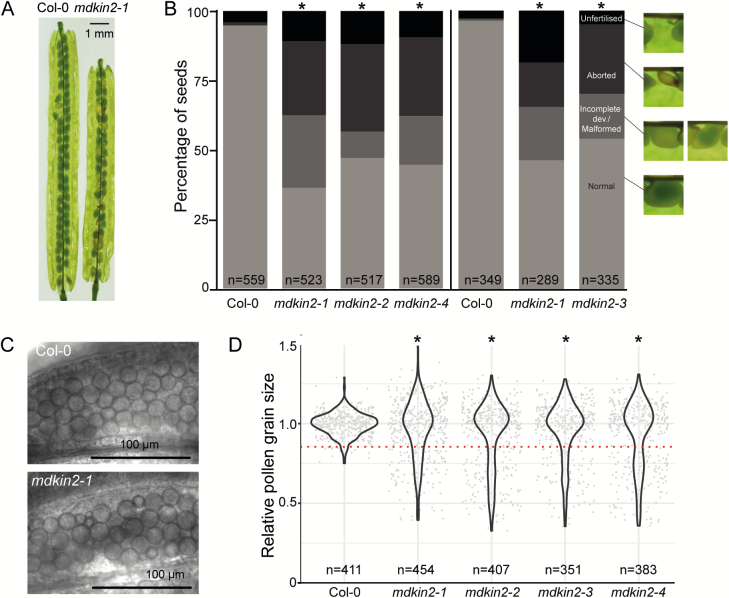
MDKIN2 affects seed and pollen development. Over 50% of seeds are abnormal or ovules are not fertilized in the siliques of *mdkin2-1* mutants (A). Distribution of seed phenotypes in wild-type Col-0 and the *mdkin2-1* T-DNA lines (B). Seeds from 13 siliques (left of the vertical line) or six siliques (right) in total from three plants per line were scored according to phenotype. Asterisks indicate statistically significant distributions of seed categories compared with Col-0 (χ ^2^ test; *P*<<0.001). Representative seed images are taken from (A) to indicate categorization of seed development (black, unfertilized; dark grey, aborted development; mid-grey, incomplete development or malformed embryo; light grey, normal development). The vertical line denotes results from two independent experiment with plants to the left or the right of the line grown in parallel. Pollen grain size in developing anthers is more variable due to abortion of some grains (C). Bright field images of developing pollen show relatively uniformity of Col-0 pollen grain sizes compared with *mdkin2-1* pollen. More pollen grains are of relatively smaller size than expected in the *mdkin2* mutants (D). Diameters of individual pollen grains were measured in five anthers per line using ImageJ. Pollen grain size was normalized within each anther before data were combined to test whether more pollen grains than expected fell below a lower nominal size threshold (red dotted line; Fisher’s exact test; *P<1×10^–13^).

To address whether malectin domain kinesins are required in plant development, we obtained T-DNA insertion lines for each of the two *MDKIN* genes in Arabidopsis, *MDKIN1* (At1g72250) and *MDKIN2* (At2g22610; see Supplementary Fig. S2A, B for MDKIN2). Plants confirmed to be homozygous for the T-DNA insertions SALK_025966 and SALK_063550 in *MDKIN1* and *MDKIN2*, respectively, are hereafter referred to as lines *mdkin1-1* and *mdkin2-1* (see Supplementary Fig. S2C for *mdkin2-1*). Loss of mRNA transcripts was confirmed by RT–qPCR for *mdkin1-1* and *mdkin2-1* (Supplementary Fig. S2D). Both T-DNA insertion lines have a normal vegetative phenotype (see Supplementary Fig. S2E for *mdkin2-1*), but *mdkin2-1* plants have reproductive development abnormalities. In homozygous *mdkin2-1* plants, ~60% of seeds failed to develop normally (Fig. 1A, B). The defects are variable, ranging from aborted and abnormal seeds to ovules that appear unfertilized.

Given that multiple T-DNA insertions are possible in the SALK lines, three further T-DNA insertion lines were acquired to confirm that reduced seed set was linked to the T-DNA insertion in *MDKIN2*. All lines (*mdkin2-2*, *mdkin2-3*, and *mdkin2-4*) were confirmed by PCR genotyping to have a homozygous T-DNA insertion in *MDKIN2* (Supplementary Fig. S2C). Categorization of seed developmental phenotypes in dissected siliques of the four *mdkin2* T-DNA insertion lines indicated that >50% of developing seeds in each line had abnormal phenotypes (Fig. 1B). Consistent seed defects in four T-DNA insertion lines of *MDKIN2* confirmed that the seed development phenotype is associated with loss of the malectin domain kinesin rather than other T-DNA insertions in these lines.

**Fig. 2.**
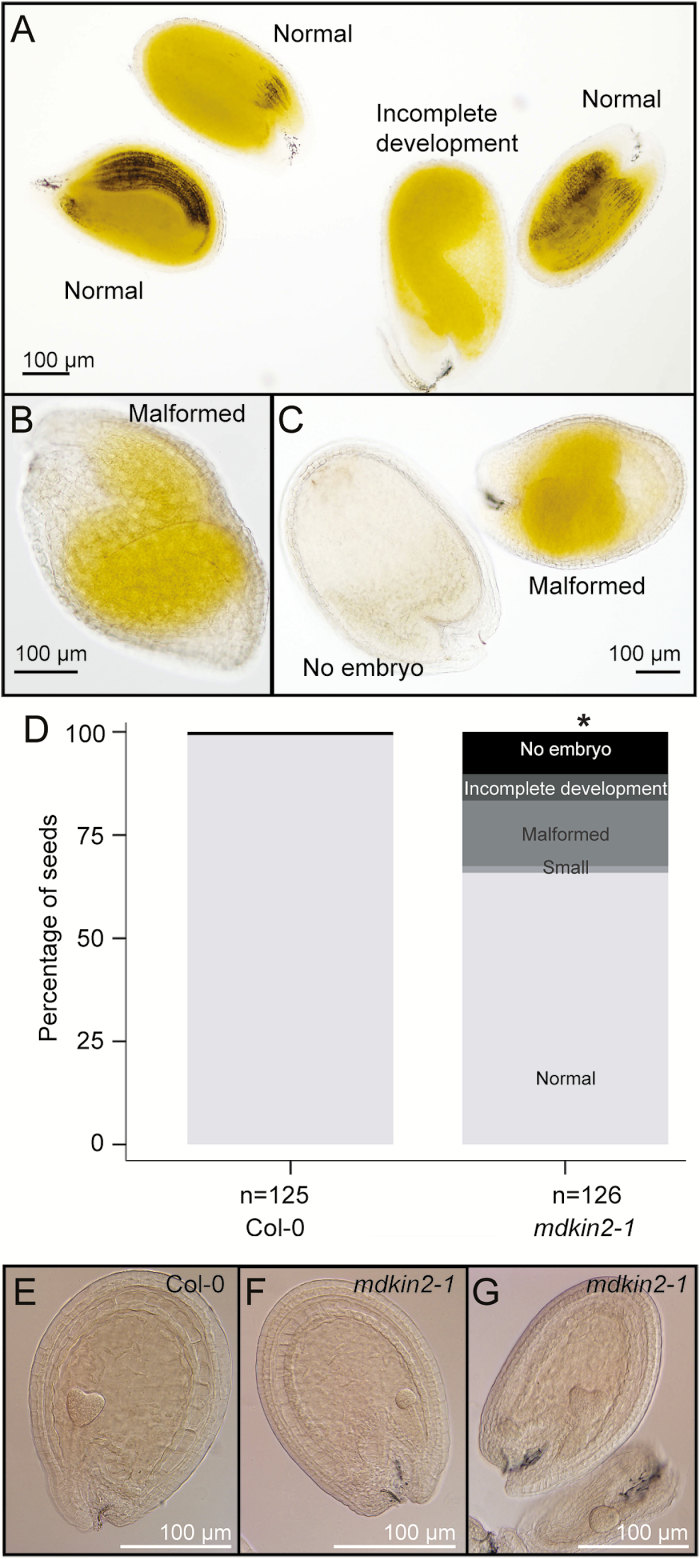
Developmental defects in cleared *mdkin2-1* seeds. Seeds from mature *mdkin2-1* siliques at 13 d after hand pollination of emasculated flowers were cleared in Hoyer’s solution then visualized by Nomarski microscopy on an Olympus BX51 microscope (A–D). Phenotypic variation includes embryos with incomplete development (A), malformed embryos with the cotyledons towards the antipodal end of the seed (B) or with indistinct organ structure (C; right seed), and seeds with a fully developed endosperm but no visible embryo (C; left seed). Quantification of defects in cleared *mdkin2-1* seeds (D). Seeds from three Col-0 and three *mdkin2-1* siliques at 13 d after hand pollination of emasculated flowers were cleared in Hoyer’s solution then visualized by Nomarski microscopy. Phenotypic variation included embryos that were smaller than normal, malformed embryos, embryos with incomplete development, and seeds with no embryo visible within the endosperm. The asterisk indicates a statistically significant distribution of seed categories compared with Col-0 (χ ^2^ test; *P*<<0.001). Note that compared with the phenotypic distribution in dissected mature siliques (Fig. 1B), unfertilized ovules and most early aborted seeds are not represented in this assay. Clearing of seeds was also performed at 6 d after hand pollination of emasculated flowers (E–G). Compared with wild-type Col-0 seeds (E), most *mdkin2-1* seeds had normal development; however, the development of some *mdkin2-1* embryos was delayed (F), and endosperm development was incomplete in other seeds, probably leading to seed abortion (G; lower seed).

Developmental issues in the embryo or the endosperm can cause seed defects. To study endosperm and embryo development in *mdkin2-1*, seeds from mature siliques were cleared in Hoyer’s solution. While some embryos were fully developed, consistent with the normal appearance of ~40% of seeds in dissected siliques, other embryos were incompletely developed or malformed, while in other seeds no embryo could be located within the endosperm (Fig. 2A–E). Clearing of seeds at an earlier stage of development (6 d after hand pollination of emasculated flowers) followed by Nomarski microscopy confirmed defects in both the embryo and the endosperm of a subset of *mdkin2-1* seeds (Fig. 2E–G). The broad range of phenotypes indicates that it is not a specific developmental step that is impaired in *mdkin2* seeds, but rather that abnormalities appear stochastically.

Some developmental defects such as mild embryo malformation may not prevent germination or further development of the seedling, therefore we used a genetic approach to confirm the frequency of survival of homozygous *mdkin2-1* progeny from heterozygous *mdkin2-1* parents as compared with wild-type progeny. Genotyping of progeny from heterozygous *mdkin2-1* plants indicated a non-Mendelian segregation ratio as expected, with homozygous *mdkin2-1* progeny recovered at 37% of the frequency of wild-type progeny (χ ^2^ test *P*<0.001; Supplementary Fig. S3). The survival rate is thus similar to the frequency of normal embryos in *mdkin2-1* mutant siliques. Genotyping indicated no haploinsufficiency as the ratio of homozygous wild-type progeny to heterozygous progeny was 1:2, although sensitivity may be reduced when using a genotyping assay compared with examining seed phenotypes due to the lower number of individuals that can be tested.

Beyond defects in seed development, we observed a high rate of aborted pollen grains in mature anthers, which are much smaller than normal pollen grains (Supplementary Fig. S4A–C). Staining with fluorescein diacetate, a compound that is converted to a fluorescent derivative by esterases in viable cells, indicated that shrunken pollen grains are not viable (Supplementary Fig. S4D; Heslop-Harrison and Heslop-Harrison, 1970). Subsequent inspection of developing anthers identified higher variability in immature pollen grain size compared with the relative uniformity of grain size in wild-type Col-0, with many *mdkin2-1* pollen grains smaller than those of wild-type Col-0 plants (Fig. 1C,D). The pollen grain phenotype was consistent across the four *mdkin2* T-DNA lines (Supplementary Fig. S5). In contrast to the pollen grains, developmental defects of female gametophytes were not consistently found.

### MDKIN2 has a partial maternal effect on seed development

**Fig. 3.**
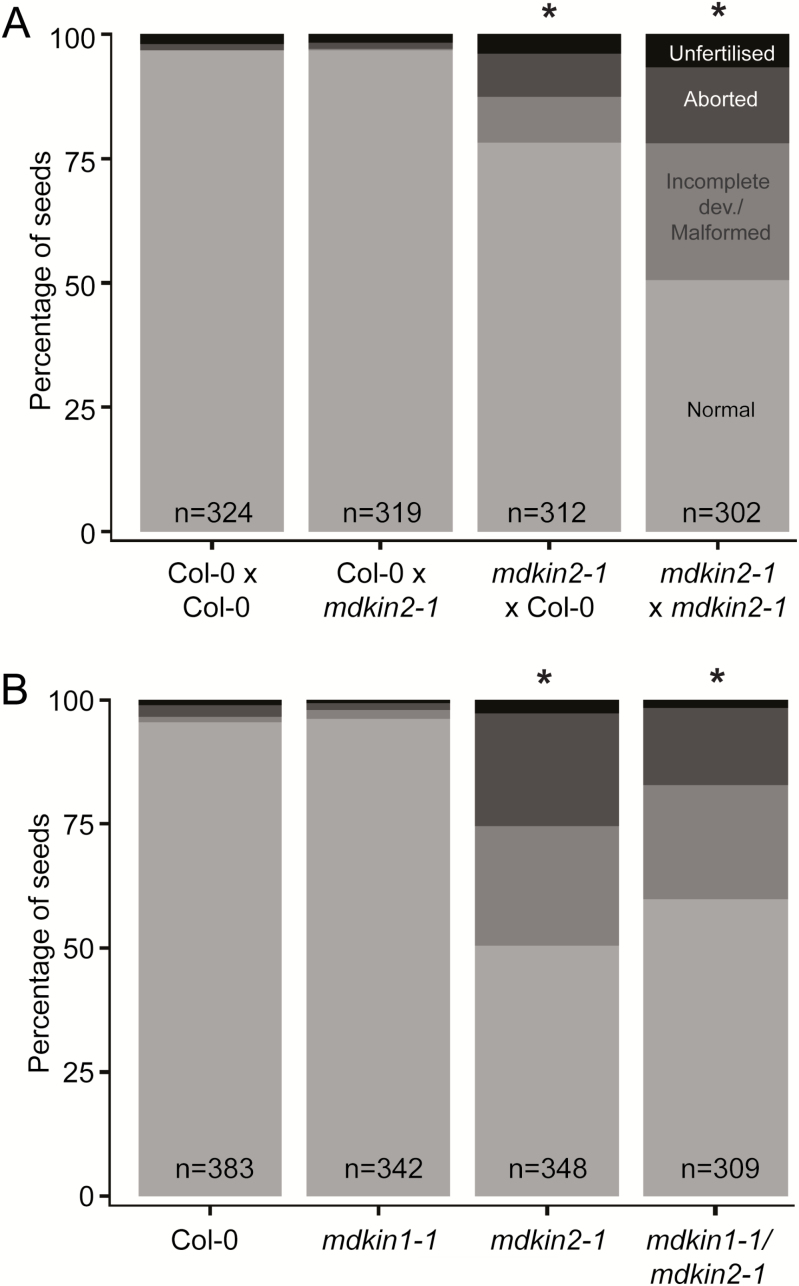
Genetic dissection of the *mdkin2-1* seed phenotype. Reciprocal crosses between Col-0 and *mdkin2-1* demonstrate a partial maternal effect on the seed development phenotype (A), while there is no functional redundancy between MDKIN1 and MDKIN2 (B). Seeds from six siliques from three plants per line or cross were scored according to phenotype, with seed categories as per Fig. 1B. Asterisks indicate statistically significant distributions of seed categories compared with the Col-0×Col-0 cross for (A) or Col-0 for (B). χ ^2^ test; *P*<<0.001. χ ^2^ test of *mdkin2-1* versus *mdkin1-1/mdkin2-1* gives *P*=0.05.

Given the broad range of seed abnormalities, we further investigated the genetic basis of this phenotype. Seed defects can arise from impaired communication between the male and female gametophytes prior to or during fertilization. In this scenario, transmission of the developmental defect would occur exclusively through the male or female line, while in contrast post-fertilization defects would be indicated by recessive inheritance of the phenotype through both germlines. To address whether the seed phenotype arises from pre- or post-fertilization events, we reciprocally crossed wild-type Col-0 plants with *mdkin2-1* mutants, along with control hand pollination of emasculated Col-0 and *mdkin2-1* plants. These crosses show no effect on seed phenotype when *mdkin2-1* is the pollen donor, but a partial effect when the mutant is the female parent (Fig. 3A). The result from the reciprocal crosses is suggestive of an effect on seed development predominantly post-fertilization rather than an effect on communication between gametes prior to or during fertilization.

As the seed developmental defects were not fully penetrant, we hypothesized that there may be functional redundancy between the two MDKIN proteins, with MDKIN2 having a partially dominant role during seed development. Therefore, we crossed the *mdkin1-1* T-DNA insertion line to *mdkin2-1*, and identified F_2_ plants that were homozygous for *mdkin2-1* and heterozygous for *mdkin1-1*. If MDKIN1 and MDKIN2 were functionally redundant during seed development, we would expect a non-Mendelian segregation ratio in the progeny for MDKIN1. Under our growth conditions, we found no functional redundancy between the two genes (Supplementary Fig. S6), with the *mdkin1-1* T-DNA inherited in a ratio of 1:2:1 of wild-type, hemizygous, and homozygous progeny. The absence of a role for MDKIN1 in seed development was confirmed through examination of the seed development phenotype in double homozygous *mdkin1-1 mdkin2-1* siliques, which have a phenotype equivalent to that of the single homozygous *mdkin2-1* plants (Fig. 3B). Pollen development was also not affected by MDKIN1 mutation (Supplementary Fig. S5). No vegetative abnormalities were observed in the *mdkin1-1* or *mdkin1-1 mdkin2-1* plants. Thus, of the two divergent malectin domain kinesins, only MDKIN2 has a clear role in pollen and seed development.

### *MDKIN2* expression and localization

**Fig. 4.**
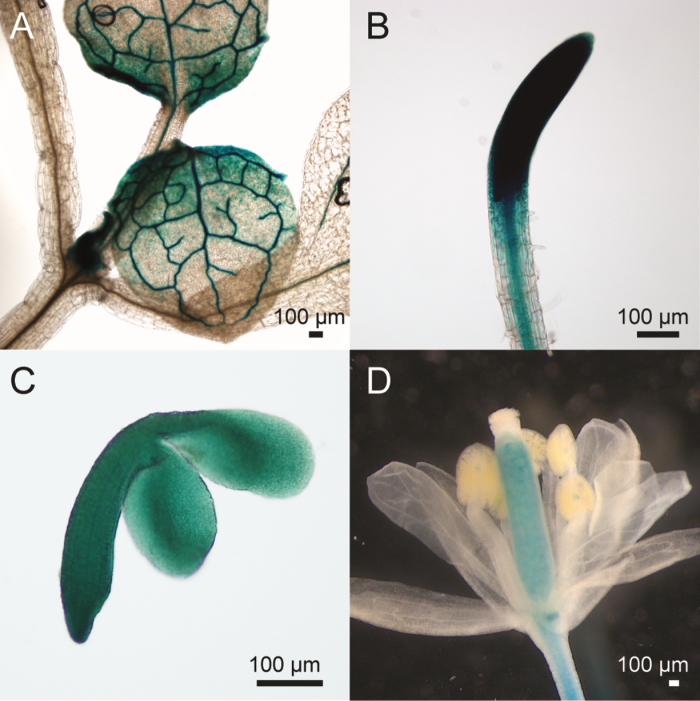
GUS reporter lines indicate *MDKIN2* expression in diverse plant tissues. In expanding leaves (A), *pMDKIN2::GUS* is expressed predominantly in the vasculature and towards the base of the leaves, while in roots (B) expression is highest in the root tip but also present in the vasculature. GUS staining is ubiquitous in developing embryos (C). In flowers (D), expression is restricted to the carpel, vasculature of the pedicel, and the junction between the filament and anther.

Although *mdkin2* mutants only have developmental defects in the male gametophytes and the seeds, the ePlant microarray data browser indicates the highest expression levels in apical meristems, especially the root tip procambium and the rib meristem of the shoot (Fucile *et al.*, 2011). To study *MDKIN2* expression at a tissue level, the *MDKIN2* promoter was fused to a *GUS* reporter gene and transformed into Col-0 plants. GUS staining revealed expression of *MDKIN2* in diverse plant tissues but predominantly in the vasculature and dividing tissues such as expanding leaves and root tips (Fig. 4A, B). In developing seeds, *MDKIN2* was expressed ubiquitously in both the endosperm (not shown) and the developing embryos (Fig. 4C), while in flowers expression was evident predominantly in the carpel (Fig. 4D). Hence *MDKIN2* expression is not restricted to the anthers and developing seeds, and is highest in dividing tissues and the vasculature.

To examine localization at a subcellular level, the *MDKIN2* promoter and coding region were fused to *tdTomato*, *mCherry*, and *GFP* reporter genes. Lines homozygous for the reporter constructs were selected in the T_3_ generation. Function of the reporter constructs was confirmed through complementation of the *mdkin2-1* seed and pollen phenotypes (Supplementary Fig. S5, S7), which demonstrates that fusion to a fluorescent protein does not impede MDKIN2 function. To co-visualize the nuclei, we crossed a pMDKIN2::MDKIN2-GFP reporter line to a plant line with the pHTR12::HTR12-mCherry reporter which marks centromeric histones (Musielak *et al.*, 2015). DAPI staining of the nuclei was not pursued as our preliminary observations indicated that it disrupts non-nuclear MDKIN2 localization, probably due to DAPI binding of the microtubules (Bonne *et al.*, 1985; Vater *et al.*, 1993).

**Fig. 5.**
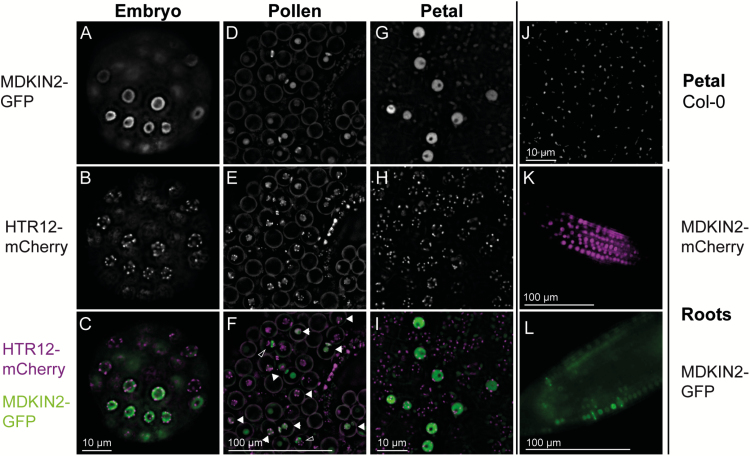
MDKIN2 is localized predominantly to the nuclei except in dividing cells. pMDKIN2::MDKIN2–GFP localization was imaged by epifluorescence microscopy along with pHTR12::HTR12-mCherry as a marker of centromeres, and hence nuclei in interphase nuclei, in embryos (A–C), developing pollen grains (D–F), and petals (G–I). Both markers were segregating in pollen; therefore, pollen grains with expression of MDKIN2 and HTR12 in the nuclei are indicated by filled arrowheads. Background autofluorescence was present in the petals and is shown for a wild-type Col-0 plant (J). A pMDKIN2::MDKIN2-mCherry marker indicates that MDKIN2 is also predominantly nuclear in the root zone of cell elongation (K). In the root zone of cell division, MDKIN2 localization is occasionally in a band perpendicular to the axis of the root (L), which is interpreted as localization during cell division. Non-nuclear MDKIN2 localization is also seen in two pollen grains undergoing mitosis I as indicted by open arrowheads in (F).

Epifluorescence microscopy of pMDKIN2::MDKIN2-GFP in globular and heart-stage embryos, and pollen, shows that MDKIN2 is predominantly localized in the nucleus (Fig. 5A–F; Supplementary Fig. S8). We also examined MDKIN2 subcellular localization in developing petals where there are fewer cell layers and in roots where there is a spatially defined region of cell division. In petals and in the root zones of expansion and differentiation (thus in interphase/G_0_), mCherry or GFP signal is localized in many cells to the nucleus, with a darker region indicative of the nucleolus particularly evident in petals (Fig. 5G–L). Fluorescence becomes less frequent and fainter further away from the zone of cell division, indicating turnover of the MDKIN2 protein. Expression can also be found in selected cells further along the developed root. The nuclei of cells within nascent lateral roots strongly express MDKIN2, as do some cells within the vascular bundle (Supplementary Fig. S9). The latter may be xylem or phloem pole pericycle cells that are about to divide or have recently undergone periclinal division during secondary growth of the root (Miyashima *et al.*, 2013) or pericycle founder cells during stages I or II of lateral root formation (Casimiro *et al.*, 2003). The observed localization patterns concur with the prediction of nuclear localization signals in the protein sequence and prior microarray data on MDKIN2 expression that indicate strong expression in meristems.

Given the partial maternal effect on seed development in *mdkin2-1* mutants, we further examined expression of MDKIN2–GFP in gametes as different parental expression of MDKIN2 could explain the maternal effect. In mature pollen grains (*n*=18) and in germinated pollen tubes (*n*=23) containing HTR12–mCherry, and where we would expect 50% presence of the MDKIN2–GFP reporter construct, no GFP fluorescence was observed. Likewise, no GFP signal was present in emasculated mature ovules of the same plant line (*n*=28). Thus, MDKIN2 is not expressed in mature male or female gametophytes such that maternal expression pre-fertilization does not contribute to the partial maternal effect on seed development. This is likely to be due to a lack of cell division in mature gametophytes.

As gametophytic expression of MDKIN2 does not contribute to the maternal effect, parental imprinting could affect expression in the early stages of embryo and endosperm development. To test whether parental imprinting affects MDKIN2 expression timing, we reciprocally crossed wild-type Col-0 or *mdkin2-1* to *mdkin2-1* pMDKIN2::MDKIN2-GFP pHTR12::HTR12-mCherry plants in combinations where seed development is not affected. Maternally and paternally derived MDKIN2 expression could be observed in both nuclei when ovules at 9.5 h post-pollination contained two nuclei within the endosperm (Supplementary Fig. S10A; stage IIb–c; Faure *et al.*, 2002). Expression is also present in embryos at the two-cell stage regardless of the direction of the cross (Supplementary Fig. S10B). As MDKIN2–GFP expression is evident before developmental defects arise in the embryo or endosperm, we conclude that the partial maternal effect does not arise due to a delay in MDKIN2 expression when it is inherited from the maternal linage.

### MDKIN2 localization during cell division

At least a third of Arabidopsis kinesins are up-regulated in expression during mitosis, indicating a likely function in the cell cycle (Menges *et al.*, 2005; Vanstraelen *et al.*, 2006). Cell division roles for a broad range of kinesins are further supported by localization of 60% of moss kinesins to mitotic structures (Miki *et al.*, 2014). In the latter data set, the closest moss homologues of MDKIN2 (Kinesins 14-IIIa and IIIb, which belong to the MDKIN1 clade) were shown to relocalize from the microtubules of the midzone in prometaphase, to the intraspindle, polar regions, and intraphragmoplast during later stages of mitosis (Miki *et al.*, 2014). However, localization for any member of the angiosperm-specific MDKIN2 clade during the cell cycle has not been reported.

**Fig. 6.**
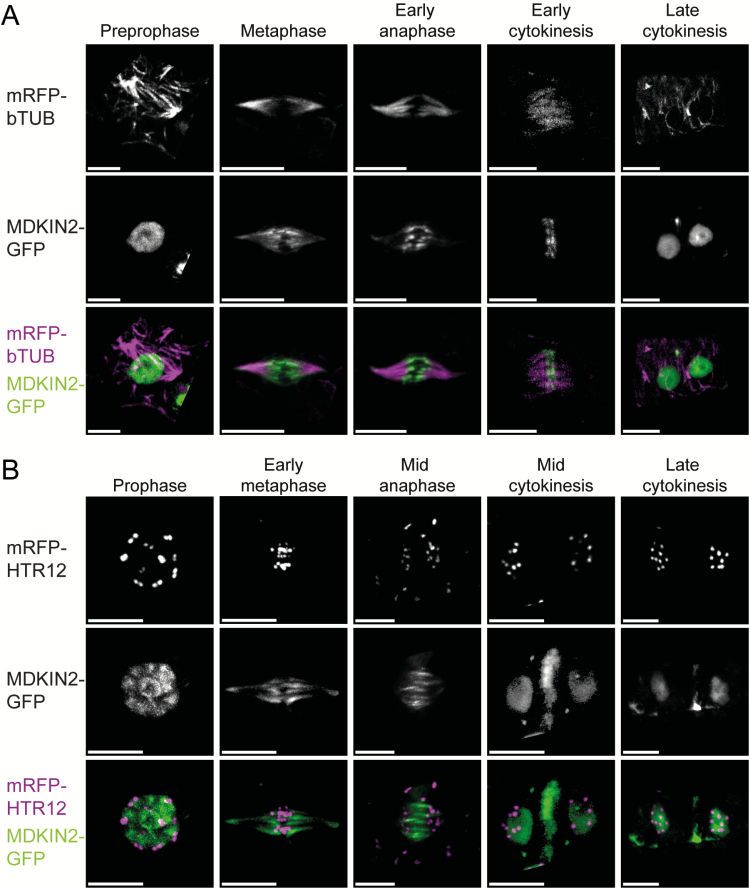
MDKIN2 is localized to the spindle and phragmoplast in embryos. Using a pUBQ1::mRFP-βTUB to mark microtubule arrays (A) and pHTR12::HTR12-mCherry as a centromere marker (B) in conjunction with pMDKIN2::MDKIN2-GFP, cells at key stages of division were imaged by spinning disk confocal microscopy in dissected heart- and torpedo-stage embryos. All scale bars are 5 μm

In the zone of cell division in roots and in developing pollen, we observed that MDKIN2 localization is not always nuclear (Fig. 5), which we hypothesized was due to different localization during cell division. To study localization of MDKIN2 during cell division, we crossed lines expressing homozygous pMDKIN2::MDKIN2-GFP that complements an *mdkin2-1* mutation to plants expressing pUBQ1::mRFP-βTUB6 (Ambrose *et al.*, 2011) or pHTR12::HTR12-mCherry. Co-expression of MDKIN2–GFP and mRFP–βTUB6 allows observation of MDKIN2 localization during cell division in relation to structures formed of microtubules, such as the preprophase band and spindle. In developing heart- and torpedo-stage embryos dissected from immature seeds, we observed that at preprophase β-tubulin is localized to the preprophase band while MDKIN2 remains in the nucleus (Fig. 6A; Supplementary Videos S1, S2). In metaphase, with centromeres aligned at the cell division zone and β-tubulin forming a spindle, MDKIN2 localizes to the tubulin spindle (Fig. 6A, B; Supplementary Video S3). MDKIN2 movement is dynamic during anaphase, with localization shifting from a spindle-like structure broadly overlapping with the tubulin spindle, to localization at the midzone as the centromeres move further towards the poles (Fig. 6A, B; Supplementary Video S4). MDKIN2 then forms a plate-like structure across the phragmoplast at the cell division zone during early cytokinesis (Fig. 6A). As cytokinesis progresses, MDKIN2 exhibits lateral expansion in a ring-like structure (Fig. 6B; Supplementary Fig. S11), while also relocalizing to the daughter nuclei (Fig. 6A, B).

**Fig. 7.**
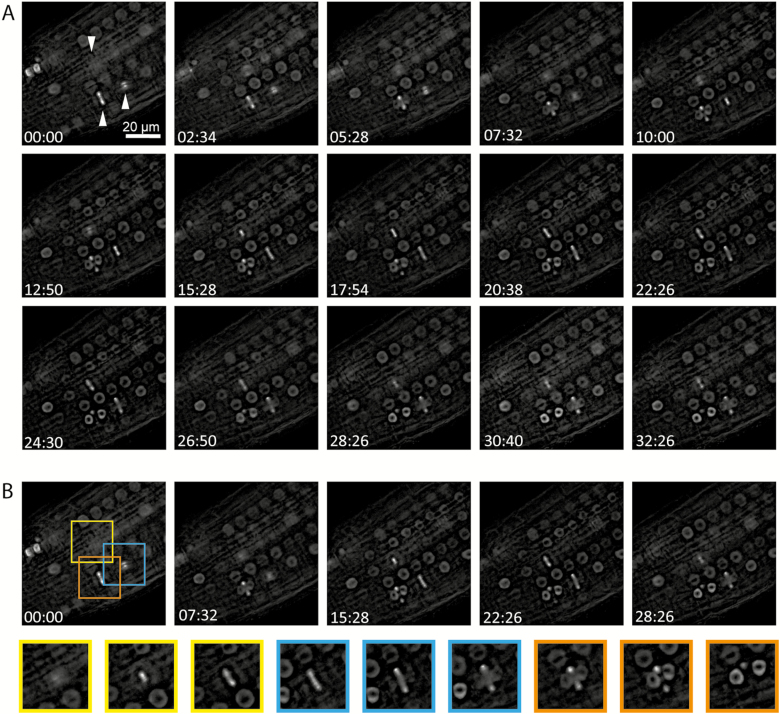
MDKIN2 localization changes during the cell cycle. Time-lapse series of dividing cells in the root tip of an *mdkin2-1* mutant complemented with pMDKIN2::MDKIN2-GFP over 32 min (A). Of particular note are the three cells indicated by arrowheads. Images from these three cells taken from five images across the time series in (A) demonstrate the changing localization of MDKIN2 during cell division (B).

To address whether the localization of MDKIN2 differs in tissues where no developmental phenotype is evident, static cells from roots were also imaged from the *mdkin2-1* pMDKIN2::MDKIN2-GFP pHTR12::HTR12-mCherry and *mdkin2-1* pMDKIN2::MDKIN2-GFP pUBQ1::mRFP-βTUB6 lines (Supplementary Fig. S12), as well as developing seed coats (Supplementary Video S1), petals (Supplementary Video S2), and actively dividing cells in roots of *mdkin2-1* pMDKIN2::MDKIN2-GFP (Fig. 7). Epifluorescence microscopy was used due to increased stability of the fluorescence signals compared with rapid photobleaching by spinning disk confocal microscopy, especially evident in roots. To test comparability of expression patterns between imaging systems, epifluorescence imaging of developing embryos was also performed (Supplementary Fig. S13). Patterns of localization observed by both epifluorescence and confocal imaging and shown in Supplementary Figs S12 and S13, but not Fig. 6, include accumulation at the spindle poles during early anaphase, and occasional diffuse localization within the cytoplasm during anaphase. In seed coats, the MDKIN2 protein is often rapidly removed from nuclei after cell division is complete (Supplementary Video S5), while in petals relocalization to the nuclei is not as distinct (Supplementary Video S6). Otherwise, localization of MDKIN2 during cell division is relatively consistent across plant tissues, and is similar to that of the moss homologues but with additional accumulation within the nuclei during interphase and preprophase.

A preliminary assessment of MDKIN1 localization in the root cell division zone using an mCitrine reporter construct was also undertaken. MDKIN1 is expressed in a smaller subset of cells in the root tip, with localization either diffuse throughout the cytoplasm or concentrated at the centre of the cell division zone in the phragmoplast (Supplementary Fig. S14). Thus, in contrast to MDKIN2, MDKIN1 expression and localization is more restricted, with overlap of the two MDKINs at cell division cytoskeletal structures only possible at the phragmoplast, although co-localization of the MDKIN proteins will need to be verified in plants co-expressing the two reporter constructs.

## Discussion

Here we have identified a role for a malectin domain-containing kinesin during pollen and seed development in Arabidopsis. Despite only having a phenotypic effect during these two discrete stages of development, MDKIN2 is expressed in other plant organs, especially in zones of cell proliferation. Its localization pattern during cell division is complex; in early phases, such as metaphase, MDKIN2 localization tracks closely to that of the tubulin spindle, while at later stages MDKIN2 appears to function at the phragmoplast midzone. Furthermore, between cell divisions, the kinesin is localized to the nucleus or, as seen in the seed coat, is turned over and removed from the cells.

A partial maternal effect was noted for the seed phenotype of the MDKIN2 mutant. This does not appear to be due to differences in expression in the gametes as MDKIN2 expression was absent in germinated or non-germinated pollen and mature ovules. Likewise, imprinting is not implicated as both maternally and paternally inherited copies of the gene are expressed very early in embryogenesis (at least by the two-nuclei stage in the endosperm and the two-cell stage of the embryo), well before developmental defects are manifest. Rather cell division in the endosperm may be more sensitive to the reduced dosage of gene expression (a third compared with a half in the embryo) and result in a maternally-derived effect of haploinsufficiency. Alternatively, as there is no obvious shift in the spectrum of developmental defects of seeds from selfed *mdkin2-1* plants to *mdkin2-1*×Col-0 crosses, the maternal environment around the developing seed could be influential (Dilkes and Comai, 2004).

The seed developmental defects in the MDKIN2 knockout mutants are pleiotropic and stochastic, sometimes affecting the endosperm and in other cases the embryo, while in pollen grains a range of sizes are observed, indicating that the effect here may also be stochastic. During seed development, we propose that the stochastic defects in the embryo may arise due to occasional disruption of mitosis in critical cells in the absence of MDKIN2. Interestingly, endosperm development involves nuclear division in the absence of cell wall formation, indicating that MDKIN2 function may be important when nuclear and cell division are not synchronized. A number of proteins are linked to pollen development, with RNAi targeting of *ANNEXIN5* (*ANN5*), which encodes a protein that putatively links cell membranes, the actin cytoskeleton, and Ca^2+^ signalling, resulting in pollen abortion from the bicellular stage (Zhu *et al.*, 2014). The *Ann5* RNAi lines have 20–40% pollen grain abortion, with the appearance of the mature pollen grains similar to that of *mdkin2* mutants. Likewise, RNAi of the putative transcription factor *TCP16* results in abortion of approximately half of the pollen grains, with grains of different sizes arising through loss of DNA at the unicellular stage of pollen development (Takeda *et al.*, 2006). It was proposed that TCP16 could regulate the expression of cell cycle genes during male gametogenesis, with loss of TCP16 triggering programmed cell death. MDKIN2 could conceivably function in a similar way to either ANN5 or TCP16, with loss leading to structural defects, initiation of programmed cell death, or both. As for endosperm development, non-standard cell division is also observed during pollen development. One sperm cell retains a cytoplasmic connection with the vegetative nucleus (Lalanne and Twell, 2002), and the two sperm cells are separated only by a thin layer of extracellular matrix rather than a cell wall dense with cellulose and callose (McCue *et al.*, 2011). Microtubules may be responsible for maintaining the overall shape of the sperm cells and the cytoplasmic projection to the vegetative nucleus (Oh *et al.*, 2010); therefore, disruption of these specialized microtubule arrays in the absence of MDKIN2 could provide an alternative mechanism underlying the pollen phenotype.

Taken together, our results suggest that the function of the MDKIN2 protein is either non-essential or partially functionally redundant in seed and pollen development, while it is non-essential in other organs. The logical candidate for functional redundancy is the second malectin domain kinesin, MDKIN1; however, we found no evidence of redundancy between MDKIN1 and MDKIN2 during reproduction or vegetative growth. This is perhaps unsurprising given the divergence of the MDKIN1 and MDKIN2 clades which occurred ~160 Mya. A number of other kinesins also have roles in cell division. Some cell cycle-related kinesins such as ATK5 (spindle organization) and KCBP (spindle and phragmoplast organization) exhibit no developmental abnormalities (Ambrose *et al.*, 2005), while others including POK1 and POK2 (which determine the plane of preprophase band formation) have severe developmental phenotypes (Lipka *et al.*, 2014). Interestingly ATK5, KCBP, and MDKIN2 have similar localization patterns at selected stages of cell division, therefore interaction between these proteins is possible. Indeed, it has been suggested that the minus-end-directed KCBP and putative plus-end-directed POK1 could act together in positioning of the phragmoplast (Müller and Jürgens, 2016). Functional redundancy between ATK5, KCBP, and MDKIN2 was, however, viewed as a less likely alternative to redundancy with MDKIN1 given the different tail domains found in each kinesin.

The function of MDKIN2 will be related to its cargo or protein binding ability. With no MDKINs previously described, some insight into potential cargos and binding ability may come from other plant malectin domain-containing proteins. Beyond the two MDKINs in Arabidopsis, the malectin domain is found in conjunction with a kinase domain in 13 receptor-like kinases of the LRR-VIII-2 family (Shiu *et al.*, 2004). No functional information is available for these proteins. However, a tandem duplication of the malectin domain (known as the malectin-like domain) is found in 17 *Cr*RLK1L, 42 LRR-1, and three LRR-1c receptor-like kinases, along with 12 further genes, many of which are probably truncated malectin-like domain receptor-like kinases (Shiu *et al.*, 2004). Arguably the best studied of the malectin or malectin-like domain receptor-like kinases are the *Cr*RLK1L family proteins FERONIA (FER), ANXUR1 (ANX1), and ANX2. Secreted peptide ligands have been identified for FER, ANX1, ANX2, and three other *Cr*RLK1L proteins (Haruta *et al.*, 2014; Ge *et al.*, 2017; Stegmann *et al.*, 2017; Gonneau *et al.*, 2018), with the binding of the RALF23 peptide to FER shown to involve the malectin domain (Xiao *et al.*, 2019). Thus, short signalling peptides provide one cargo candidate for the malectin-domain kinesins, although most signalling peptides are vesicular, membrane-associated, or apoplastic rather than localized to the nucleus.

While carbohydrate ligands for the *Cr*RLK1L proteins were initially proposed based on structural conservation with the animal malectin protein (Schallus *et al.*, 2008), only recently have the malectin-like domains of FER and other related *Cr*RLK1L proteins been demonstrated to bind pectin in the form of its building block polygalacturonic acid (Feng *et al.*, 2018; Lin *et al.*, 2018, Preprint). Recent crystal structures for ANX1 and ANX2 indicate that a ligand-binding pocket is probably formed at the interface of the two malectin domains, with carbohydrate-binding residues not conserved between animal homologues and plant *Cr*RLK1L proteins (Moussu *et al.*, 2018). As the malectin domain is present in a single copy in the MDKINs, cargo binding at such an interface could be facilitated if the MDKINs act as dimers or multimers. In support of this hypothesis, kinesin-14 proteins generally function as dimers, and some kinesin-14 proteins from the moss *P. patens* have been shown to only move along microtubules when clustered as at least dimers of dimers (Jonsson *et al.*, 2015). While kinesin-14-IIIa, an MDKIN homologue, was found to dimerize, no processivity along the microtubules was observed (Jonsson *et al.*, 2015). It should, however, be noted that the malectin domains of receptor-like kinases are extracellular, in contrast to the intracellular malectin domain of the MDKINs. If the malectin domain of MDKIN2 can likewise bind to polysaccharides, its localization to the cell division plane during cytokinesis may indicate a function in organization of cell wall components. A link between potential polysaccharide binding and MDKIN2 function in interphase nuclei of the root tips and vasculature, and when localized in the mitotic spindle, is less clear.

That MDKIN2 localizes to the nucleus in interphase, the spindle during anaphase, and the midzone of the phragmoplast during cytokinesis raises the possibility that it has diverged functions at different points in the cell cycle and in different subcellular locations. Dual localization to the nucleus and cytoplasm has been previously documented for a number of kinesins, including rice BRITTLE CULM 12 (BC12) which belongs to the evolutionarily divergent kinesin-4 family (Zhang *et al.*, 2010), a trypanosome kinesin-13 which localizes to the nucleoplasm and the mitotic spindle (Wickstead *et al.*, 2010), and rice DUAL LOCALISATION KINESIN (OsDLK) which is found on cortical microtubules and in the nucleus (Xu *et al.*, 2018). Other kinesins that function during spindle organization, chromosome condensation, and metaphase chromosome alignment include the kinesin-4 and kinesin-10 families of chromokinesins in yeast and animals, which contain a leucine zipper DNA-binding domain (Wood *et al.*, 1997; Rhee *et al.*, 2005; Zhu and Dixit, 2012). While Arabidopsis encodes three kinesin-4 proteins, only one is cell cycle regulated (Vanstraelen *et al.*, 2006), though a lack of canonical nuclear localization signal has led to the suggestion that it may not function as a chromokinesin (Zhu and Dixit, 2012). However, with no putative DNA-binding domain, MDKIN2 seems unlikely to act as a chromokinesin, leaving the question open as to whether chromokinesins exist in Arabidopsis and whether MDKIN2 could co-localize or function as a heterodimer with a chromokinesin.

Clearly further research will be required to define the function or functions of MDKIN2 during various stages of the cell cycle, in different subcellular regions and in diverse tissues. Why loss of MDKIN2 disproportionally affects development of pollen and seeds while having no evident effect on root growth needs to be addressed, along with a careful dissection of how developmental defects in the pollen, endosperm, and embryo arise. Furthermore, identification of the cargo or other macromolecules bound by MDKIN2 will go a long way to inform the function of this unusual small family of plant-specific kinesins which has persisted for almost half a billion years.

## Supplementary data

Supplementary data are available at *JXB* online.

Fig. S1. Comparative phylogenetic analysis of the MDKIN proteins in plants.

Fig. S2. Multiple independent T-DNA lines of *MDKIN2* do not significantly affect vegetative growth.

Fig. S3. Mutation of *MDKIN2* distorts the segregation ratio.

Fig. S4. Pollen grain development is aborted in *mdkin2-1* plants.

Fig. S5. Pollen grain development is affected in multiple *mdkin2* lines and is complemented by fluorescently tagged MDKIN2.

Fig. S6. *MDKIN1* mutation does not have an additive effect on mutant allele inheritance in the *mdkin2-1* background.

Fig. S7. The *mdkin2-1* seed phenotype can be complemented by fluorescently tagged MDKIN2.

Fig. S8. MDKIN2 is expressed in the nuclei of globular embryos.

Fig. S9. MDKIN2 is expressed in lateral root meristems and the vascular system.

Fig. S10. MDKIN2 is expressed during early cell divisions of the endosperm and embryo post-fertilization.

Fig. S11. MDKIN2 localization during cytokinesis.

Fig. S12. MDKIN2 localization during cell division in roots and seed coats.

Fig. S13. MDKIN2 is localized to the spindle and phragmoplast in embryos.

Fig. S14. MDKIN1 localization in the root tip.

Video S1. MDKIN2 localization relative to tubulin during preprophase.

Video S2. MDKIN2 localization relative to tubulin during anaphase.

Video S3. MDKIN2 localization relative to the centromeres during preprophase.

Video S4. MDKIN2 localization relative to the centromeres during metaphase.

Video S5. MDKIN2–GFP localization during cell division in the seed coat.

Video S6. MDKIN2–GFP and centromere localization during cell division in developing petals.

Table S1. Genotyping primers.

Table S2. RT-PCR and RT–qPCR primers.

Table S3. Cloning primers.

eraa023_suppl_supplementary_video_S1

eraa023_suppl_supplementary_video_S2

eraa023_suppl_supplementary_video_S3

eraa023_suppl_supplementary_video_S4

eraa023_suppl_supplementary_video_S5

eraa023_suppl_supplementary_video_S6

eraa023_suppl_supplementary_figures_S1_S14_tables_S1-S3

## Abbreviations

βTUB: β-tubulin
*Cr*RLK1L: *Catharanthus roseus* Receptor-Like Kinase 1-Like
HTR12: centromeric histone H3
MDKIN: MALECTIN DOMAIN KINESIN
UBQ: ubiquitin
