# Supplementary material for: A malectin domain kinesin functions in pollen and seed development in Arabidopsis
Source: J Exp Bot. 2020 Jan 17;71(6):1828–41. doi: 10.1093/jxb/eraa023 (PMC7094084; doi:10.1093/jxb/eraa023)
Supplement: eraa023_suppl_supplementary_figures_S1_S14_tables_S1-S3 [file eraa023_suppl_supplementary_figures_s1_s14_tables_s1-s3.pdf]

Supplementary Figures, Videos and Tables

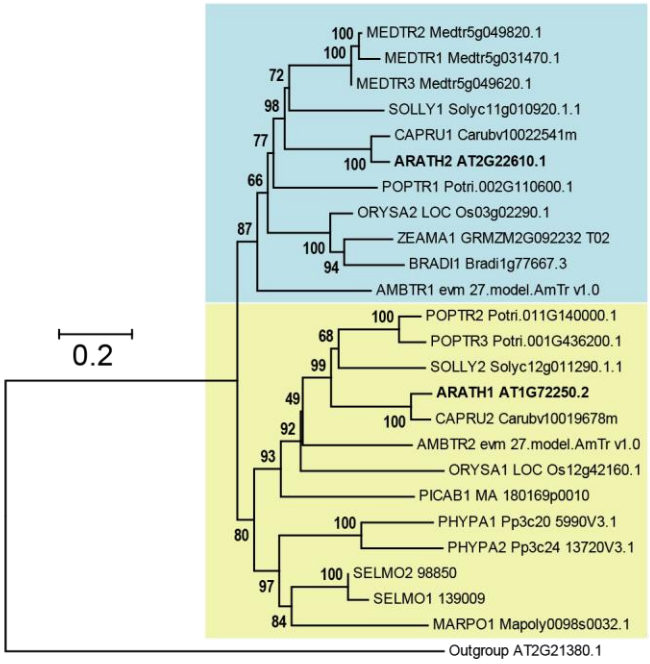

Figure S1:

Comparative phylogenetic analysis of the MDKIN proteins in plants. Sequences were aligned using ClustalX (Larkin *et al.*, 2007). The evolutionary history was inferred using a Neighbour-Joining phylogenetic tree generated with the software MEGA5.2 (Tamura *et al.*, 2011, Saitou and Nei, 1987). The percentage of replicate trees in which the associated taxa clustered together in the bootstrap test (1000 replicates) is shown next to each branch. MDKIN proteins were identified from *A. thaliana* (ARATH), *M. truncatula* (MEDTR), *S. lycopersicum* (SOLLY), *C. rubella* (CAPRU), *P. trichocarpa* (POPTR), *B. distachyon* (BRADI), *O. sativa* (ORYSA), *Z. mays* (ZEAMA), *A. trichopoda* (AMBTR), *P. abies* (PICAB), *S. moellendorffii* (SELMO), *P. patens* (PHYPA), and *M. polymorpha* (MARPO). The kinesin protein At2g21380 from *A. thaliana* was used as an outgroup. MDKIN2 (At2g22610) is within the angiosperm specific clade highlighted in blue while MDKIN1 (At1g72250) is in the clade highlighted in yellow.

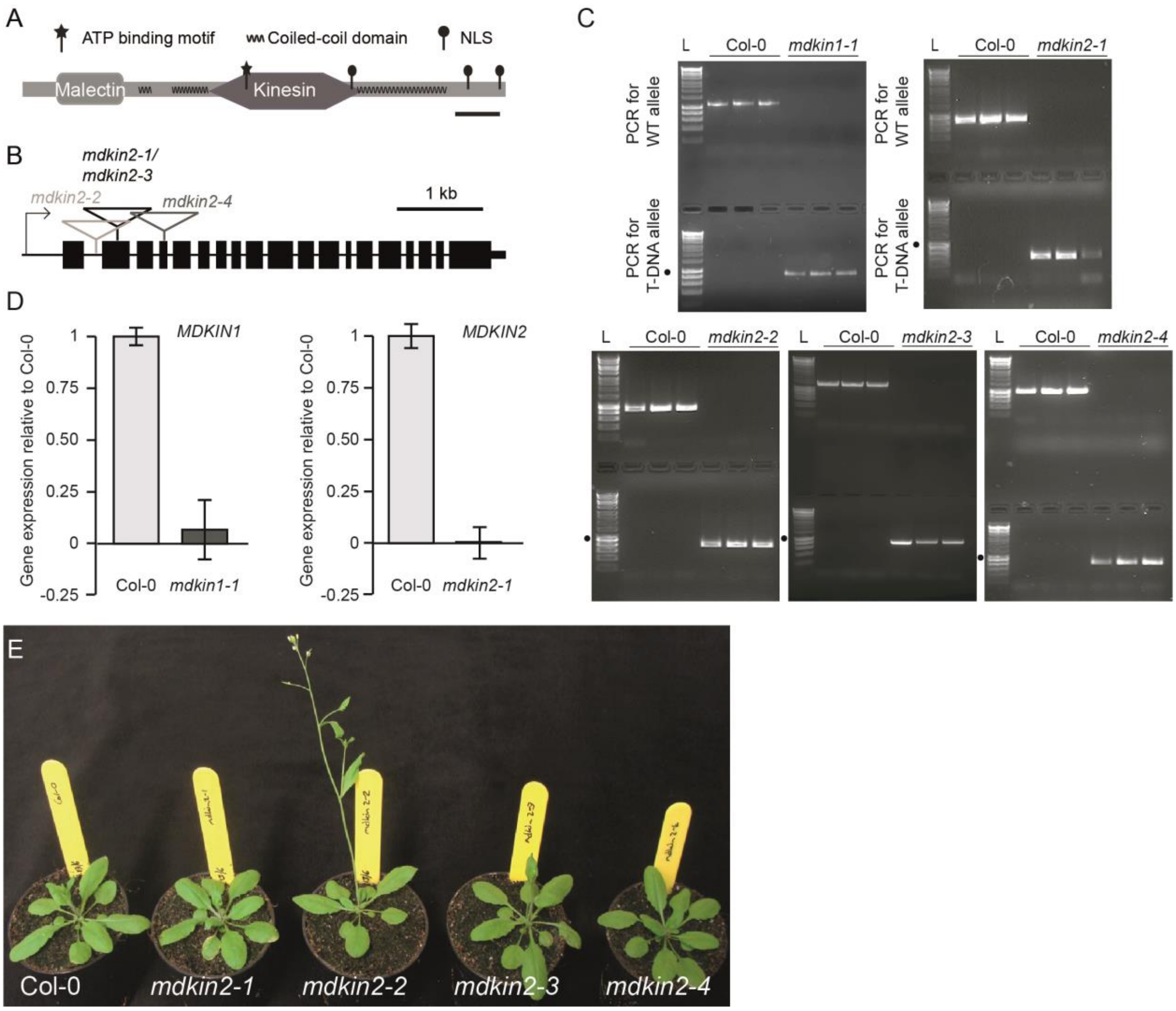

Figure S2:

Multiple independent T-DNA lines of MDKIN2 do not significantly affect vegetative growth. Protein domain structure of MDKIN2 **(A)**. Domain structures are as predicted by Pfam. Gene structure of MDKIN2 (At2g22610; **(B)**). Intron-exon structure is indicated and the positions of the T-DNA insertions used in this study. The T-DNA insertion in *mdkin2-3* is 68 bp 5' of the T-DNA insertion in *mdkin2-1*. Presence of the T-DNA insertion was confirmed by PCR for *mdkin1-1*, *mdkin2-1*, *mdkin2-2, mdkin2-3* and *mdkin2-4* **(C)**. Dots indicate 1 kb ladder band. Loss of *MDKIN1* and *MDKIN2* mRNA transcripts in the *mdkin1-1* and *mdkin2-1* mutants respectively was confirmed by RT-qPCR (mean of three biological replicates ± standard deviation; **D**). Actin was used as a control. Oligonucleotide sequences are listed in Tables S1 and S2. Vegetative phenotype of the four MDKIN2 T-DNA lines compared to wildtype Col-0 **(E)**. Plants were grown in long day conditions for 28 days at 23ºC.

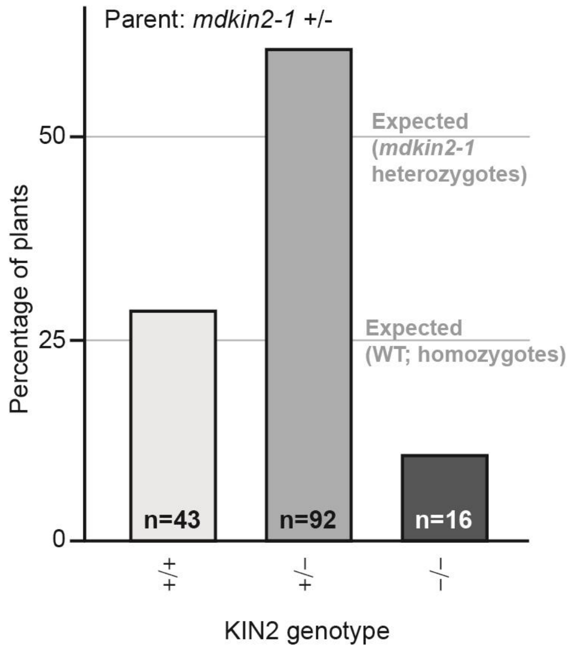

Figure S3:

Mutation of *MDKIN2* distorts the segregation ratio. Low rates of *mdkin2-1* homozygotes amongst progeny of heterozygous mdkin2-1 plants confirm that some seeds homozygous for *mdkin2-1* fail to develop. Progeny from a plant hemizygous for the *mdkin2-1* T-DNA insertion were genotyped at *MDKIN2* (n=151). The percentage of progeny homozygous for the T-DNA insertion was lower than predicted for a Mendelian segregation ratio (χ^2 test; $p<<0.001$).

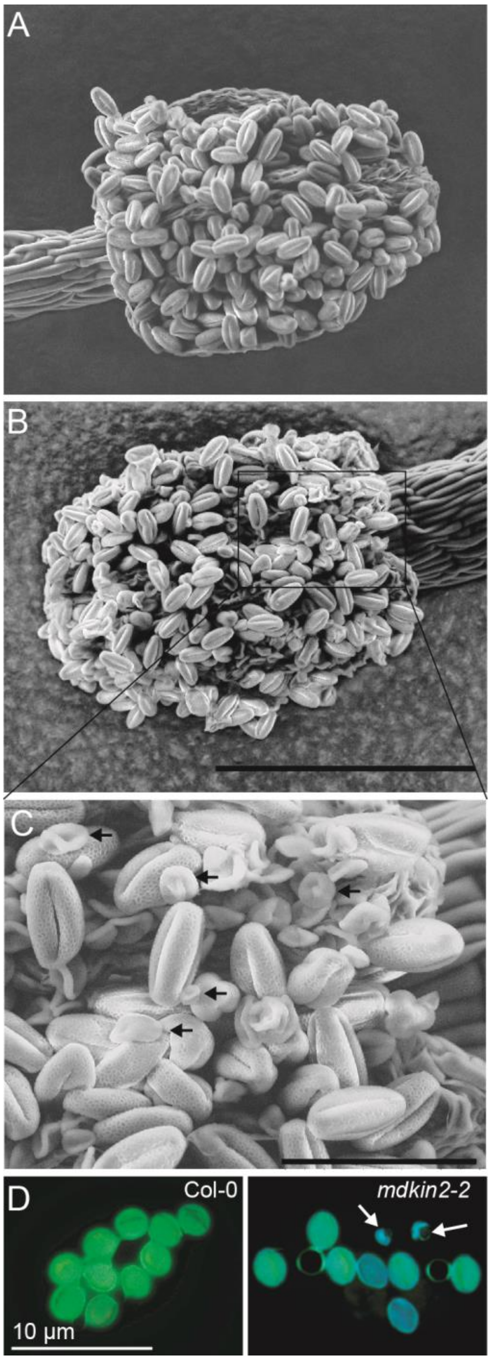

Figure S4:

Pollen grain development is aborted in *mdkin2-1* plants. SEM micrographs of Col-0 **(A)** and *mdkin2-1* anthers **(B)** demonstrate the high rate of aborted pollen grains in the mutant. Higher resolution imaging of the *mdkin2-1* anther shows that the aborted pollen grains are much smaller than the normal pollen grains as indicated by the arrows **(C)**. Scale bars are 200 µm in (A) and (B) and 50 µm in (C). FDA staining indicates loss pollen viability in aborted pollen grains of *mdkin2* mutants **(D)**. Mature pollen grains of wildtype Col-0 and the *mdkin2-1* mutant were stained for viability using fluorescein diacetate. Aborted pollen grains (indicated by arrows) remain a blue hue while viable pollen grains fluoresce yellow).

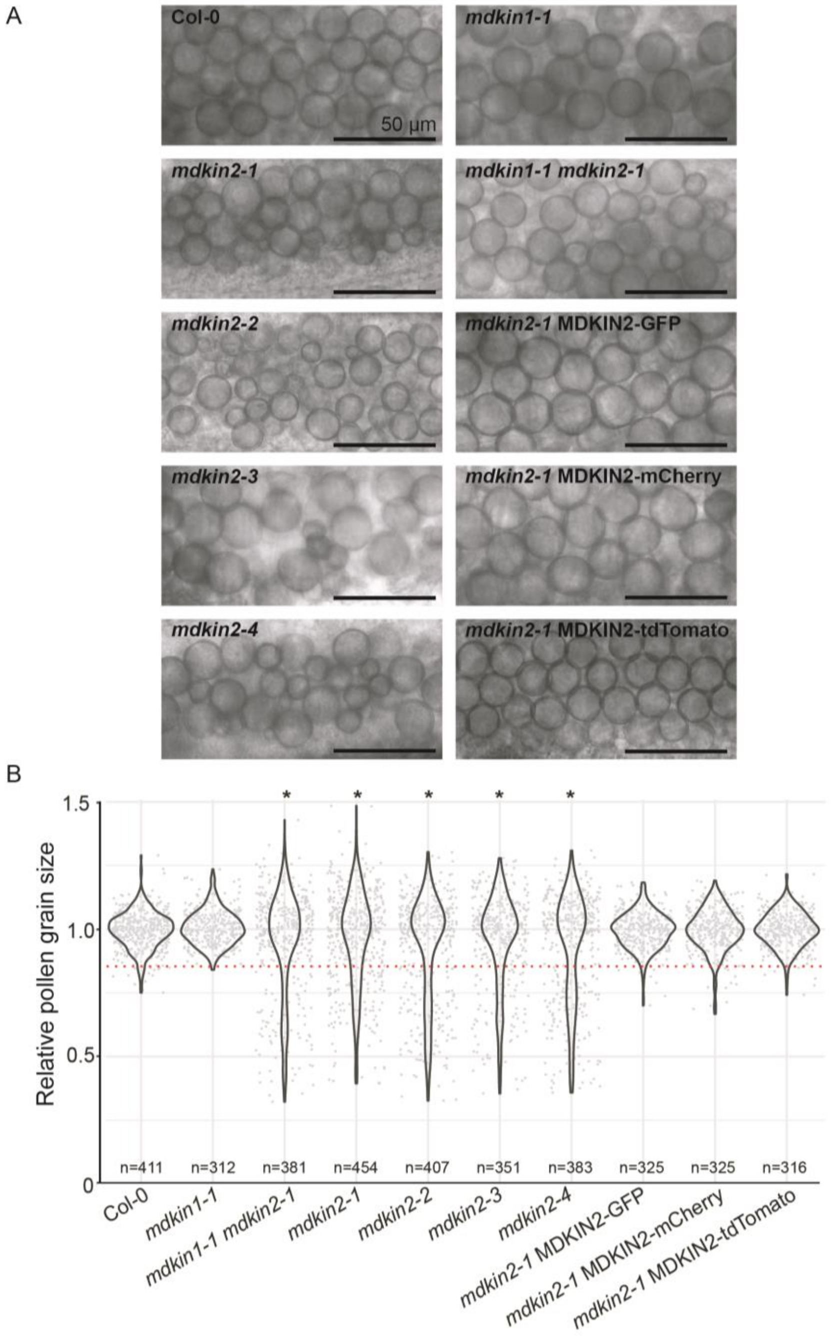

Figure S5:

Pollen grain development is affected in multiple *mdkin2* lines and is complemented by fluorescently tagged MDKIN2. Bright field images of developing pollen show uniformity of pollen grain size in WT Col-0 compared to the four *mdkin2* T-DNA insertion lines **(A)**. Pollen grain size variability is not affected in *mdkin1-1* nor is there partial functional redundancy as the phenotype is not more severe in double *mdkin1-1 mdkin2-1* mutants. Uniformity in pollen grain size is restored by complementation with fluorescently tagged MDKIN2 proteins. More pollen grains are of relatively small size than expected in the *mdkin2* mutants **(B)**. Diameters of individual pollen grains were measured in five anthers per line using ImageJ. Pollen grain size was normalised within each anther before data was combined from five anthers per genotype to test whether more pollen grains fell below a nominal size threshold (red dashed line) than expected (Fisher's exact test; * p < 1 x 10-13). Data for Col-0 and the four *mdkin2* alleles are the same as in Figure 1.

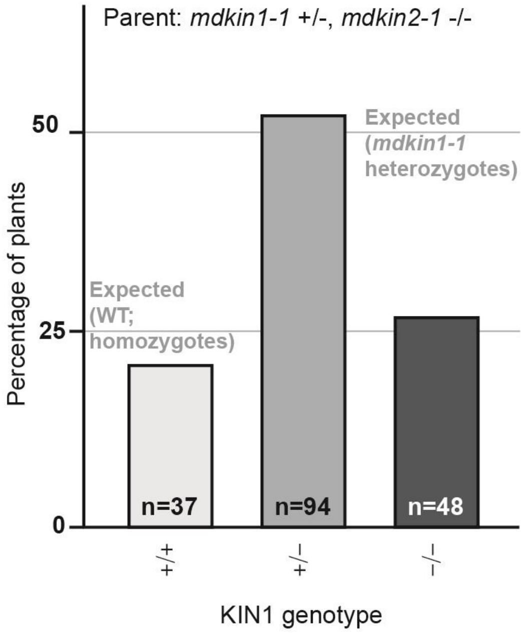

Figure S6:

***MDKIN1* mutation does not have an additive effect of on mutant allele inheritance in the *mdkin2-1* background.** To test for an additive effect from *MDKIN1* knockout, progeny from a plant homozygous for the *mdkin2-1* T-DNA insertion and hemizygous for the *mdkin1-1* T-DNA insertion were genotyped at *MDKIN1* (n=179). Seed genotype inheritance was not significantly different from a Mendelian segregation ratio (χ^2 test; p=0.41).

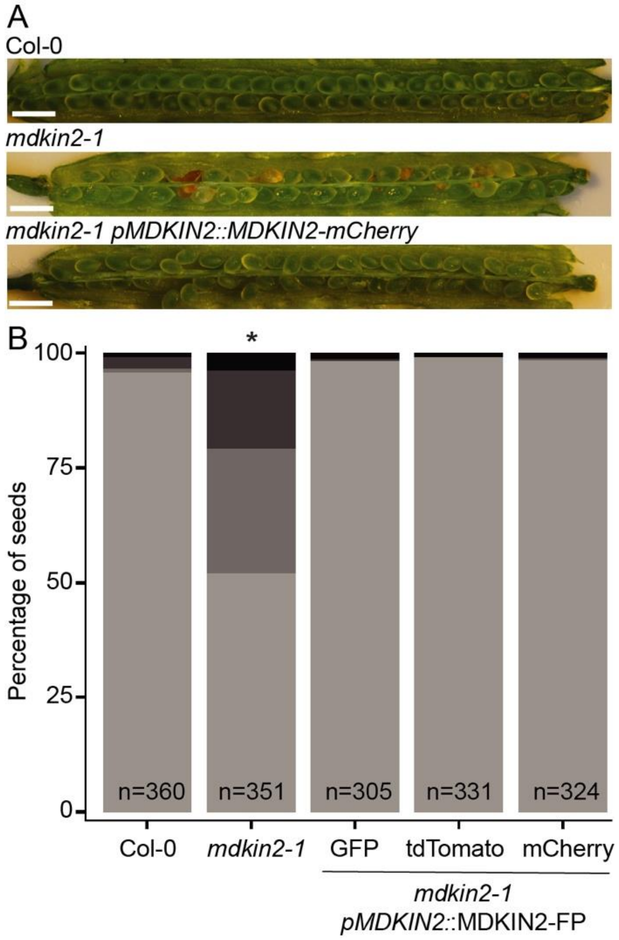

Figure S7:

The *mdkin2-1* seed phenotype can be complemented by fluorescently tagged MDKIN2. Dissected siliques from wildtype Col-0, the *mdkin2-1* mutant, and *mdkin2-1* complemented with pMDKIN2::MDKIN2-mCherry **(A)**. Scale bars = 1 mm. Seed phenotype distribution was measured for wildtype Col-0, *mdkin2-1* and *mdkin2-1* complemented with pMDKIN2::MDKIN2-GFP, pMDKIN2::MDKIN2-tdTomato or pMDKIN2::MDKIN2-mCherry constructs in homozygous T3 or T4 lines **(B)**. Six siliques per line from three plants grown in parallel were dissected, photographed and seed phenotypes scored as per Figure 1A. Categories of seed development are black: unfertilised; dark grey: aborted; mid-grey: incomplete development or malformed embryo; light grey: normal development. Asterisks indicate statistically significant distributions of seed categories compared to Col-0 (χ^2 test; $p<<0.001$).

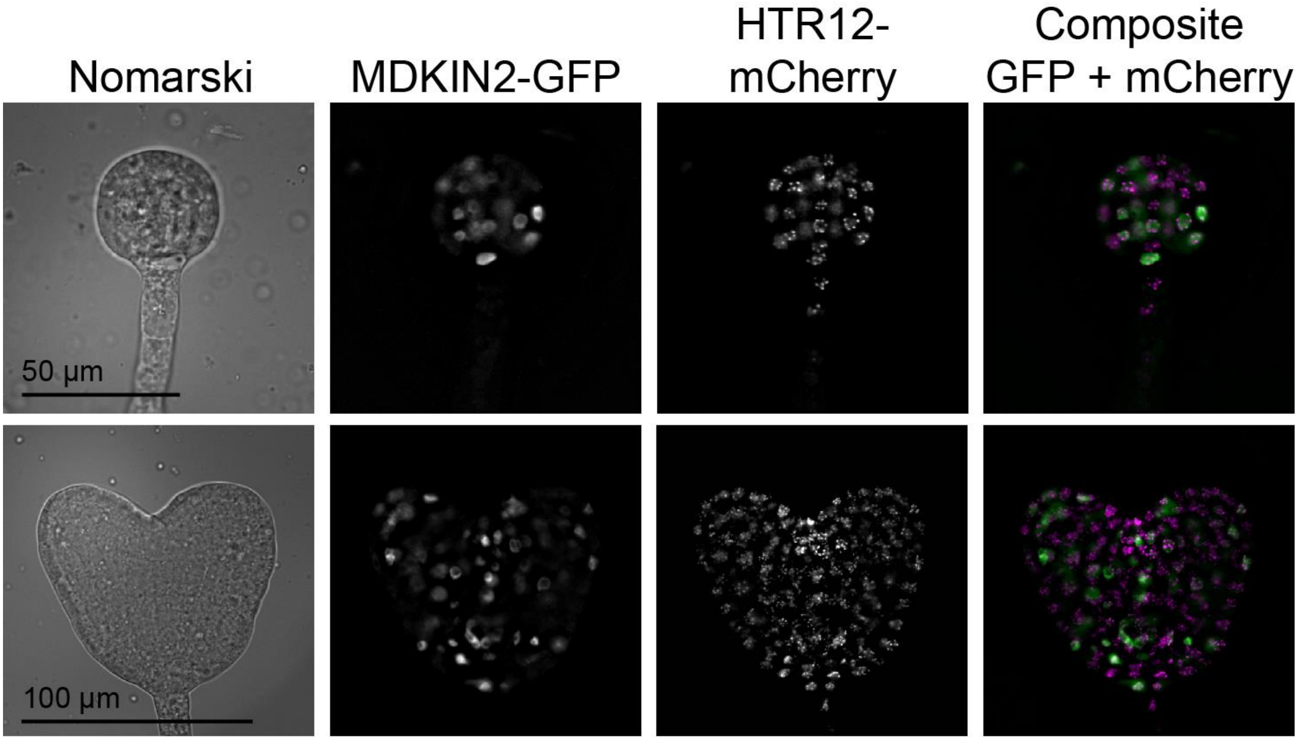

Figure S8:

MDKIN2 is localised predominantly to the nuclei of globular and heart stage embryos. MDKIN2 is also localised to nuclei within globular and heat stage embryos as visualised by pMDKIN2::MDKIN2-GFP expression. pHTR12::HTR12-mCherry is included as a centromere marker to indicate nuclear position.

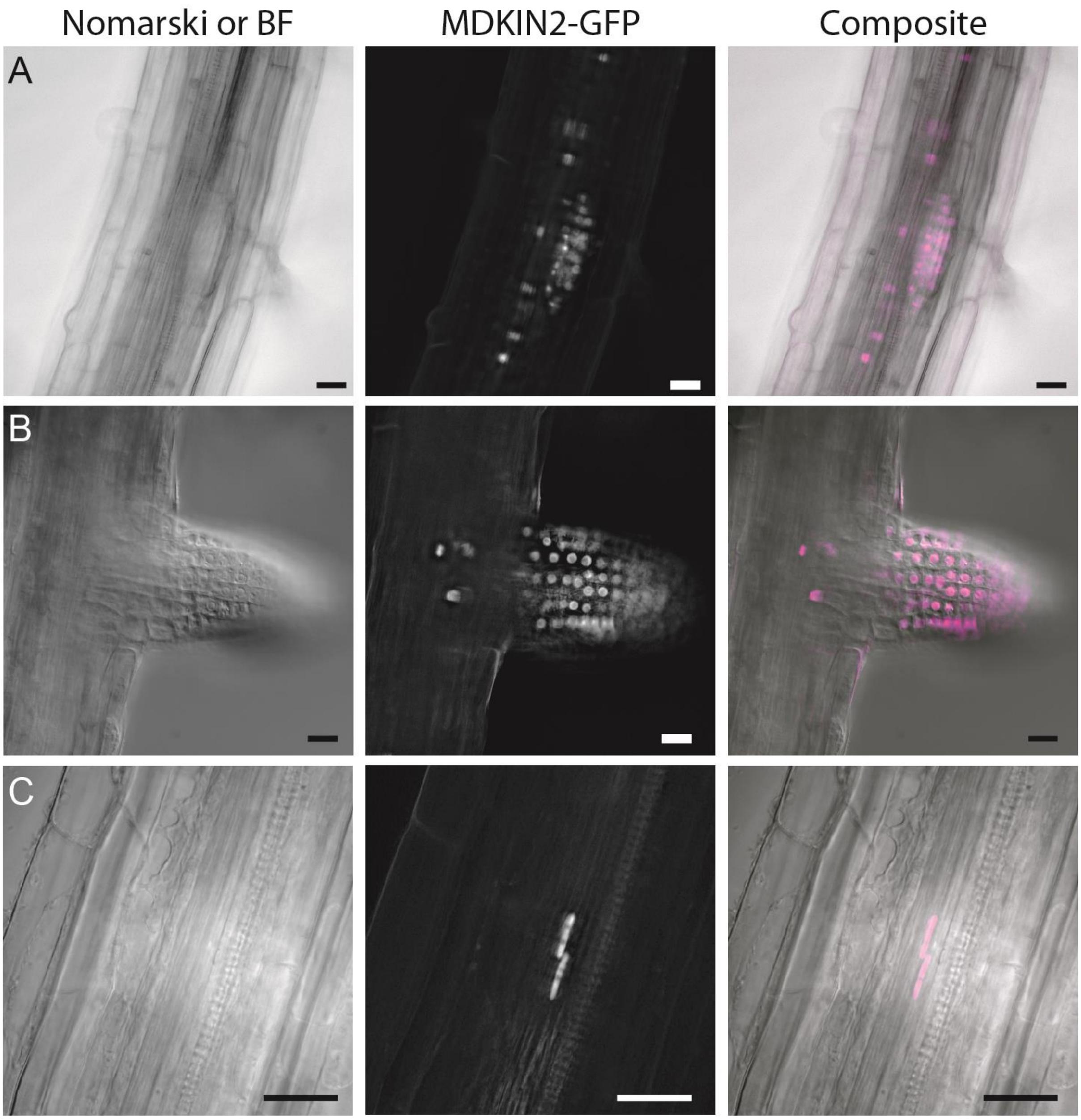

Figure S9:

MDKIN2 is expressed in lateral root meristems and the vascular system. MDKIN2 is also localised to nuclei within emerging **(A)** and older **(B)** lateral roots. Within the root vasculature, expression is limited to isolated nuclei **(C)**. Nomarski or bright field (BF) images are shown alongside epifluorescence and composite images. All scale bars are 20 μm.

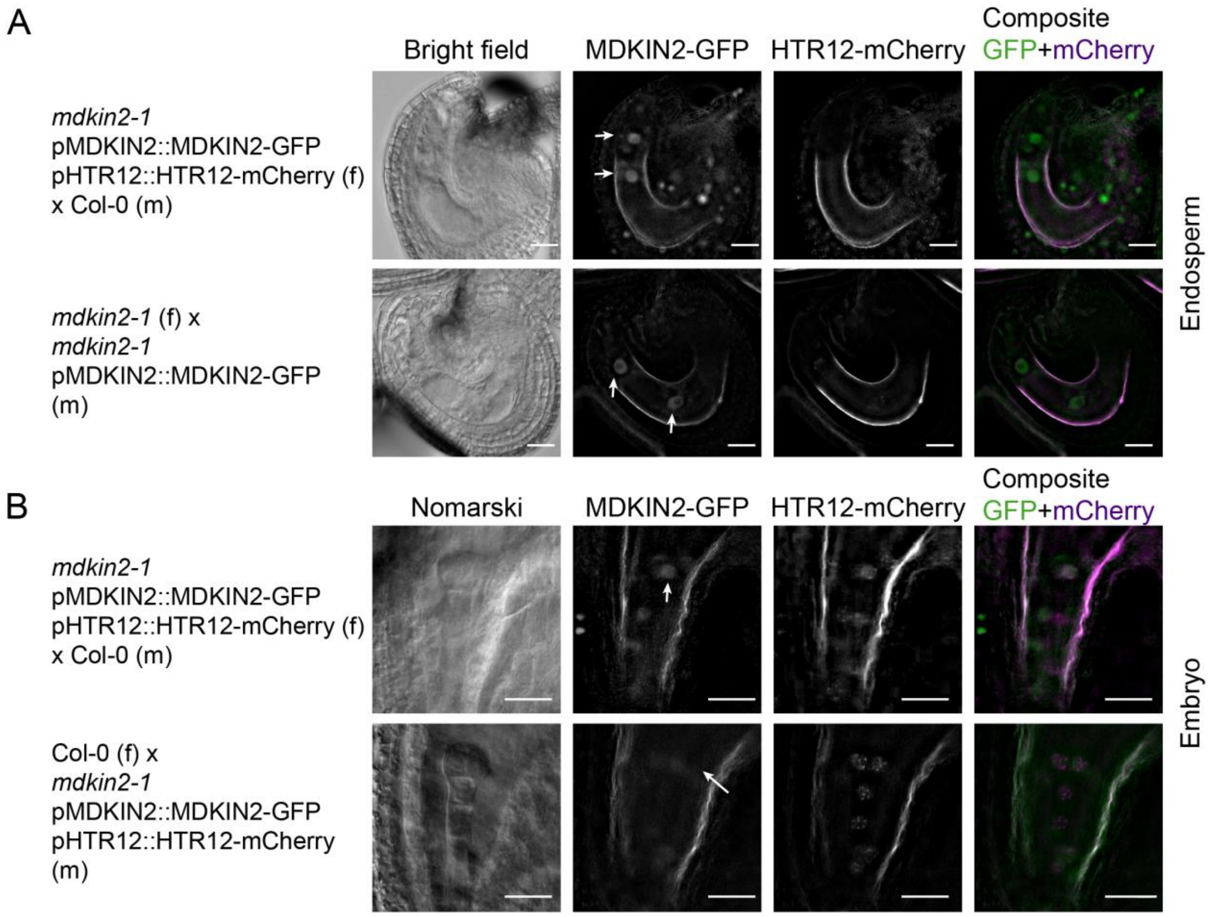

Figure S10:

MDKIN2 is expressed during early cell divisions of the endosperm and embryo post-fertilisation. Reciprocal crosses of *mdkin2-1* pMDKIN2::MDKIN2-GFP pHTR12::HTR12-Cherry and Col-0 or *mdkin2-1* were performed. Fertilised ovules were examined at 9.5 hrs or 48 hrs post-fertilisation to examine expression of MDKIN2-GFP in two-nuclei endosperm **(A)** or two-celled embryos **(B)** when inherited from the female (upper panels) or male (lower panels) parent. Arrows indicate nuclei with MDKIN2-GFP expression. All scale bars are 20 µm.

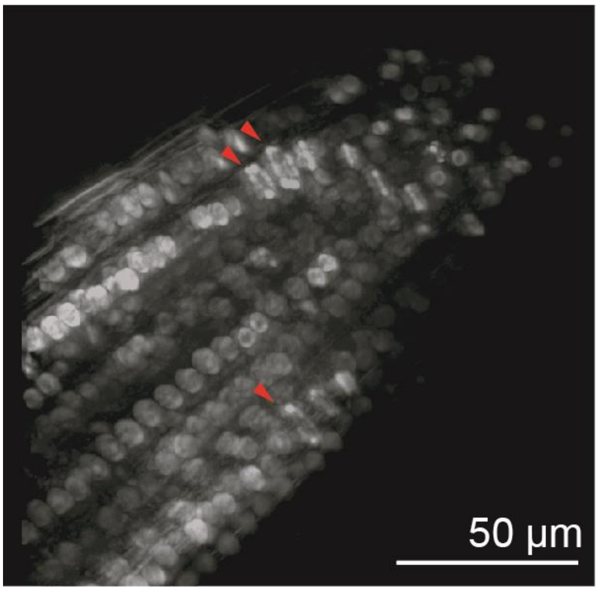

Figure S11:

MDKIN2 localisation during cytokinesis. pMDKIN2::MDKIN2-GFP localisation imaged by epifluorescence microscopy in the root tip as visualised by projection of a Z-stack. Arrows indicate rings of MDKIN2 localisation during cytokinesis.

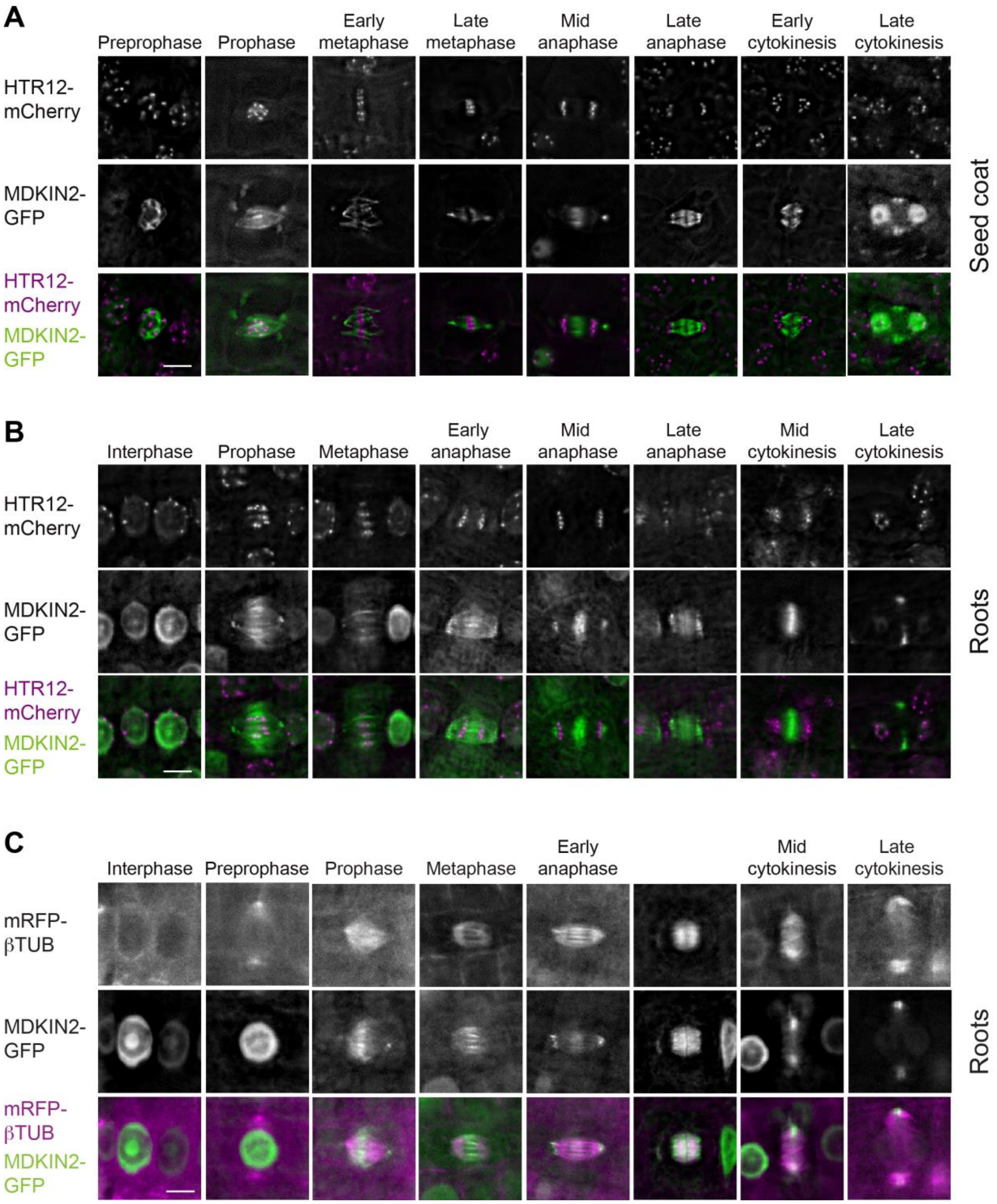

Figure S12:

MDKIN2 localisation during cell division in roots and seed coats. Using pHTR12::HTR12-mCherry as a centromere marker **(A, B)** and pUBQ1::mRFP-ßTUB **(C)** in conjunction with pMDKIN2::MDKIN2-GFP, cells at key stages of division in seed coats **(A)** and root tips **(B, C)** were imaged by epifluorescence microscopy. All scale bars are 5 μm.

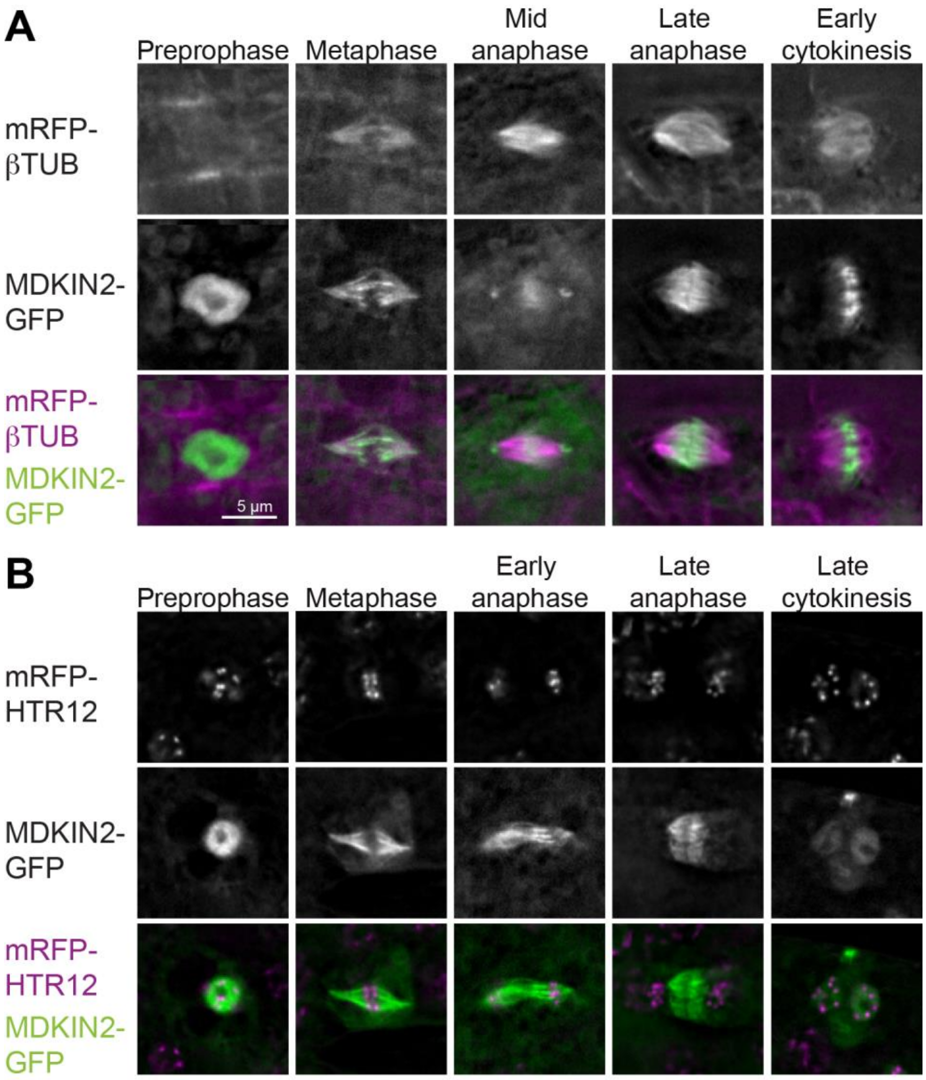

Figure S13:

MDKIN2 is localised to the nucleus, spindle and phragmoplast in embryos. Using pUBQ1::mRFP-bTUB to image microtubule arrays **(A)** and pHTR12::HTR12-mCherry to visualise centromeres **(B)** in conjunction with pMDKIN2::MDKIN2-GFP, cells at various stages of division in dissected heart and torpedo stage embryos were imaged by epifluorescence microscopy. All scale bars are 5 μm.

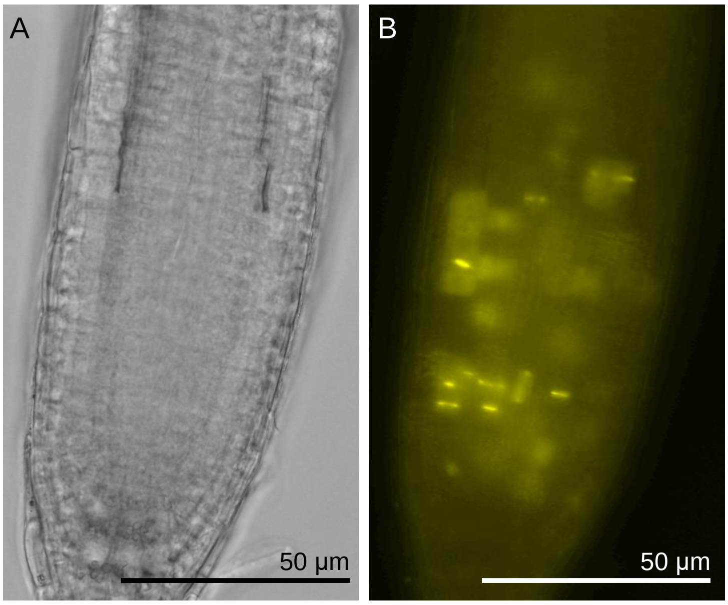

Figure S14:

MDKIN1 localisation in the root tip. Using pMDKIN1::MDKIN1-mCitrine, MDKIN1 localisation was imaged in root tips (bright field; **A**) by epifluorescence microscopy (**B**).

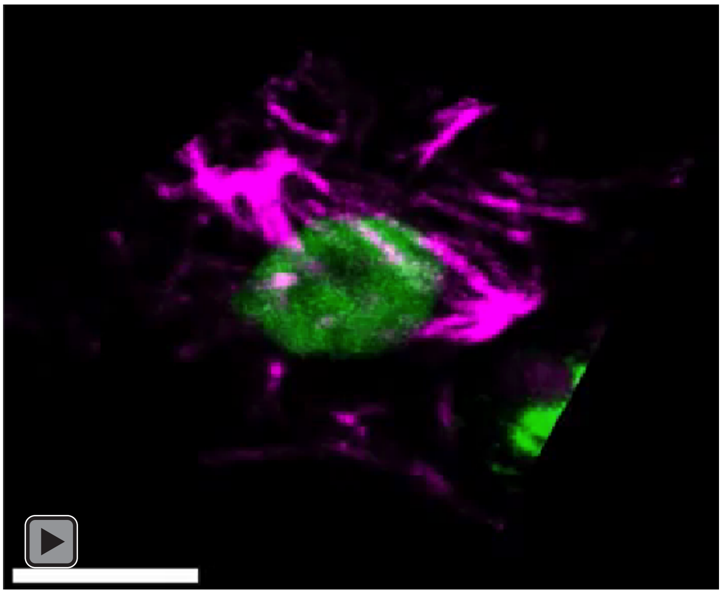

Video S1:

MDKIN2 localisation relative to tubulin at preprophase. Tubulin (pUBQ1::mRFP-bTUB) is shown in magenta and pMDKIN2::MDKIN2-GFP is shown in green. Scale bar is 5 µm.

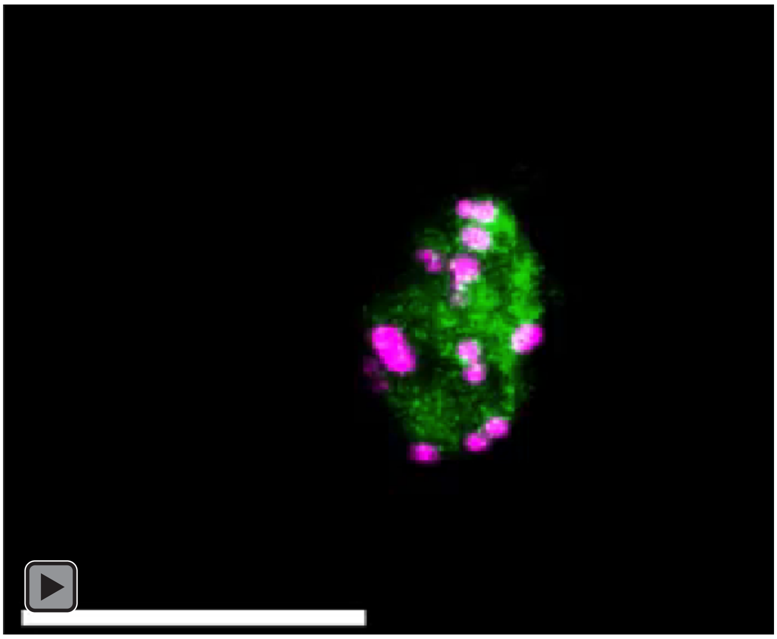

Video S2:

MDKIN2 localisation relative to the centromeres at preprophase. Centromeres (pHTR12::HTR12-mCherry) are shown in magenta and pMDKIN2::MDKIN2-GFP is shown in green. Scale bar is 5 µm.

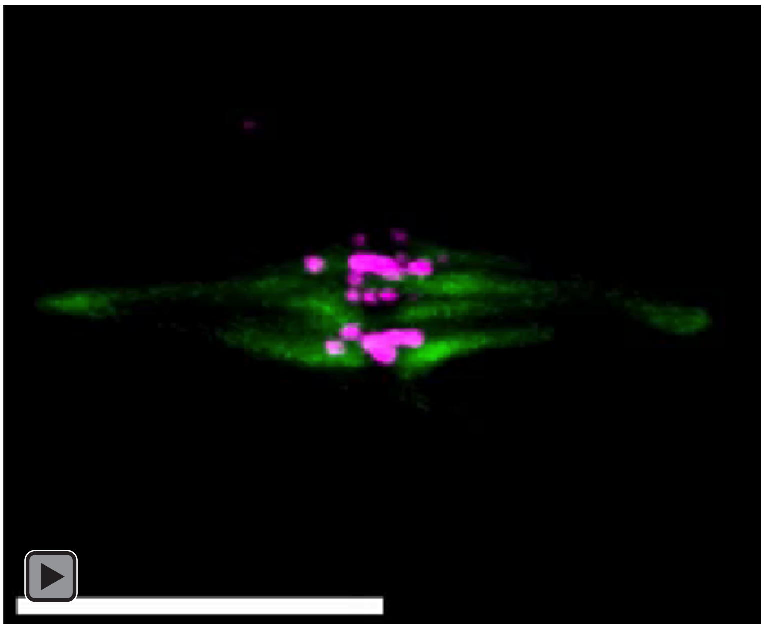

Video S3:

MDKIN2 localisation relative to the centromeres at metaphase. Centromeres (pHTR12::HTR12-mCherry) are shown in magenta and pMDKIN2::MDKIN2-GFP is shown in green. Scale bar is 5 µm.

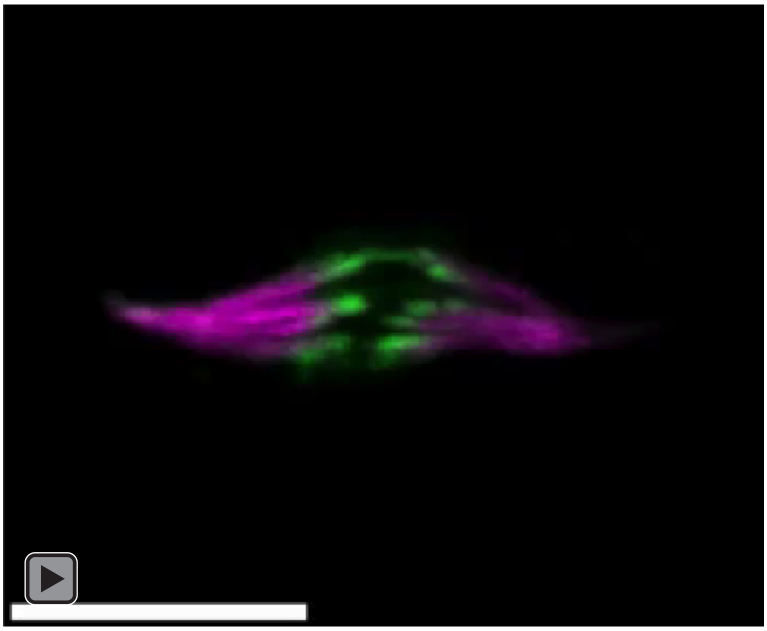

Video S4:

MDKIN2 localisation relative to tubulin at anaphase. Tubulin (pUBQ1::mRFP-bTUB) is shown in magenta and pMDKIN2::MDKIN2-GFP is shown in green. Scale bar is 5 µm.

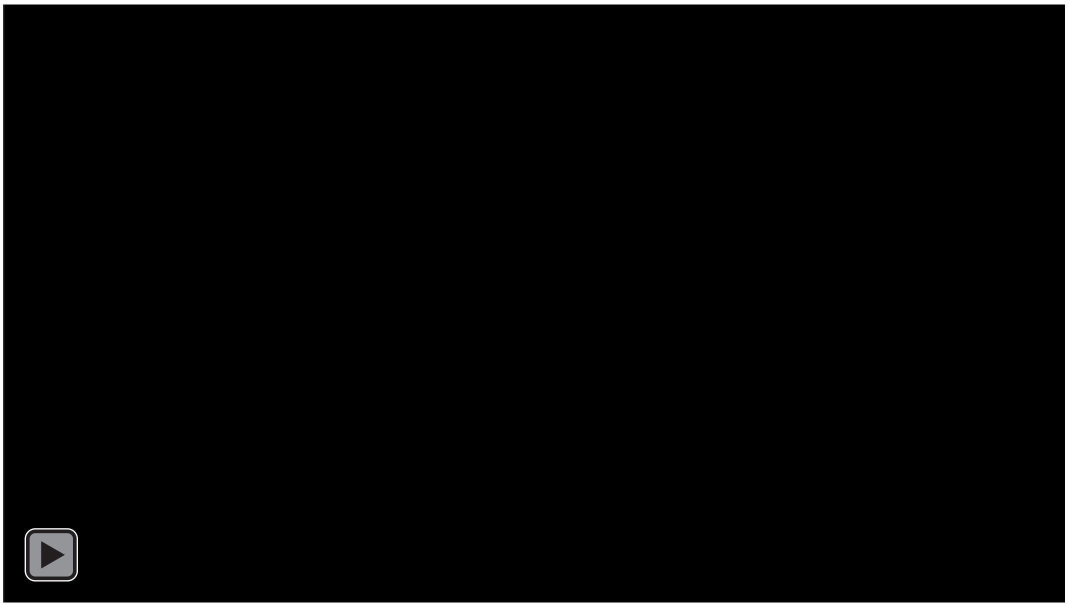

Video S5:

MDKIN2-GFP localisation during cell division in the seed coat. Autofluorescence from the embryo sac is visible in magenta **(A)**. pMDKIN2::MDKIN2-GFP is shown in green **(B)**. Merged channels are shown **(C)**. Scale bar is 25 µm.

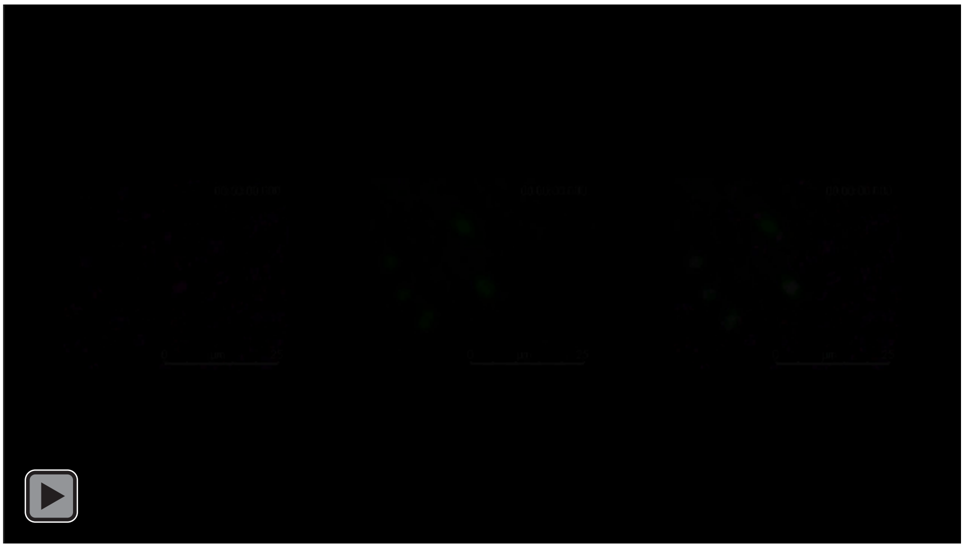

Video S6:

MDKIN2-GFP and centromere localisation during cell division in developing petals. pHTR12::HTR12-mCherry was used as centromere marker **(A)**. pMDKIN2::MDKIN2-GFP is shown in green **(B)**. Merged channels are presented **(C)**. Scale bar is 25 µm.

Supplemental Tables

Supplemental Table 1: Genotyping primers

NASC ID	T-DNA line	Gene/ Allele	WT Primer 1	WT Primer 2	T-DNA Primer (with WT primer 2)
N563550	SALK-063550	*MDKIN2*<br>*mdkin2-1*	CCGAATTCAAGACTGGTTCC	CTGTGGCGCTTTCCTAAGAC	(Salk LBb1.3) ATTTTGCCGATTTCGGAAC
N572997	SALK-072997	*MDKIN2*<br>*mdkin2-2*	CCGAATTCAAGACTGGTTCC	CTGTGGCGCTTTCCTAAGAC	(Salk LBb1.3) ATTTTGCCGATTTCGGAAC
N814463	SAIL-312_D02	*MDKIN2*<br>*mdkin2-3*	ATGGATGATGTTCAGATCGATGATAC	AGCTTTGGCTCGCATAAGTC	(Truncated SAIL LB1) AAATGGATAAATAGCCTTGCTTCC
N655068	SALK-049342C	*MDKIN2*<br>*mdkin2-4*	GATCCCTTCAACGCATTCAT	GCAAATAGGCCACTCCTGTT	(Salk LBb1.3) ATTTTGCCGATTTCGGAAC
N525966	SALK-025966	*MDKIN1*<br>*mdkin1-1*	AAGCACGAGAACACCGCTAC	TGCAGGTCCCAACTCTATCC	(Salk LBb1.3) ATTTTGCCGATTTCGGAAC
		pMDKIN2:: MDKIN2-GFP	CTACCCCGACCACATGAAGC	CTTCTCGTTGGGGTCTTTGC	
N67065		*pUBQ::mRFP-βTUB6*<br>*RFP*	GGGAGCGCGTGATGAACTTC	GGTGTAGTCCTCGTTGTGGGA	

Supplemental Table 2: RT-qPCR primers

Gene	Primer 1	Primer 2
MDKIN1 (qPCR)	TGCAGGTCCCAACTCTATCC	TGCTTCAGGATTCTCTAGGAGGA
MDKIN2 (qPCR)	TGTTCATAAATGCTGGAGGAGA	TCACCATGACCCTTAAGTCAACCAACAGGA
ACTIN2	CCAGAAGGATGCATATGTTGGTG	GAGGAGCCTCGGTAAGAAGA

Supplemental Table 3: Cloning primers

**Construct**	**Primer 1**	**Primer 2**
MDKIN2 promoter GUS fusion	CACAAAGTTTGCTGGAGGAAG	AATTATTTGGAAGCAAATCGTCTCT
MDKIN2 mCherry, tdTomato or GFP fusion	CACAAAGTTTGCTGGAGGAAG	TCTTGACCAAATCTTCTTCTTGGAGT
MDKIN1 mCitrine fusion	TCATTCGGAAAATGCAGAGA	GATCCATCGCTCTTGTTTCTGCGGTG
